# Earlier peak photosynthesis timing potentially escalates global wildfires

**DOI:** 10.1093/nsr/nwae292

**Published:** 2024-08-22

**Authors:** Gengke Lai, Jialing Li, Jun Wang, Chaoyang Wu, Yongguang Zhang, Constantin M Zohner, Josep Peñuelas, Quansheng Ge

**Affiliations:** International Institute for Earth System Sciences, Jiangsu Center for Collaborative Innovation in Geographical Information Resource Development and Application, Nanjing University, Nanjing 210023, China; Jiangsu Provincial Key Laboratory of Geographic Information Science and Technology, Key Laboratory for Land Satellite Remote Sensing Applications of Ministry of Natural Resources, School of Geography and Ocean Science, Nanjing University, Nanjing 210023, China; International Institute for Earth System Sciences, Jiangsu Center for Collaborative Innovation in Geographical Information Resource Development and Application, Nanjing University, Nanjing 210023, China; Jiangsu Provincial Key Laboratory of Geographic Information Science and Technology, Key Laboratory for Land Satellite Remote Sensing Applications of Ministry of Natural Resources, School of Geography and Ocean Science, Nanjing University, Nanjing 210023, China; The Key Laboratory of Land Surface Pattern and Simulation, Institute of Geographic Sciences and Natural Resources Research, Chinese Academy of Sciences, Beijing 100101, China; The Key Laboratory of Land Surface Pattern and Simulation, Institute of Geographic Sciences and Natural Resources Research, Chinese Academy of Sciences, Beijing 100101, China; International Institute for Earth System Sciences, Jiangsu Center for Collaborative Innovation in Geographical Information Resource Development and Application, Nanjing University, Nanjing 210023, China; Jiangsu Provincial Key Laboratory of Geographic Information Science and Technology, Key Laboratory for Land Satellite Remote Sensing Applications of Ministry of Natural Resources, School of Geography and Ocean Science, Nanjing University, Nanjing 210023, China; Huangshan National Park Ecosystem Field Scientific Observation and Research Station of the Ministry of Education, Nanjing 210023, China; Jiangsu International Joint Carbon Neutrality Laboratory, Nanjing University, Nanjing 210023, China; Department of Environmental Systems Science, Institute of Integrative Biology, ETH Zurich, Zurich 8092, Switzerland; CSIC, Global Ecology Unit CREAF-CSIC-UAB, Barcelona 08193, Spain; CREAF, Cerdanyola del Vallès, Barcelona 08193, Spain; Jiangsu International Joint Carbon Neutrality Laboratory, Nanjing University, Nanjing 210023, China; The Key Laboratory of Land Surface Pattern and Simulation, Institute of Geographic Sciences and Natural Resources Research, Chinese Academy of Sciences, Beijing 100101, China

**Keywords:** vegetation photosynthesis phenology, wildfire, climate feedback, Earth System model

## Abstract

More intense fire weather due to climate change is implicated as a key driver of recent extreme wildfire events. As fuel stock, the role of vegetation and its phenology changes in wildfire dynamics, however is not fully appreciated. Using long-term satellite-based burned areas and photosynthesis observations, we reveal that an earlier peak photosynthesis timing (PPT) potentially acts to escalate subsequent wildfires, with an increase in the global average burned fraction of 0.021% (∼2.20 Mha) for every additional day of PPT advancement. Satellite observations and Earth System modeling consistently show that this fire escalation is likely due to intensified drought conditions and increased fuel availability associated with the climate feedback arising from earlier PPT. Current fire-enabled dynamic global vegetation models can reproduce the observed negative correlation between PPT and burned area but underestimate the strength of the relationship notably. Given the continued PPT advancement owing to climate change, the bioclimatic effects of vegetation phenology change suggest a potentially pervasive upward pressure on future wildfires.

## Teaser

Satellite observations and Earth System modeling reveal that earlier peak photosynthesis timing potentially escalates global burned areas by amplifying drought conditions and increasing fuel availability.

## INTRODUCTION

Recent extreme wildfire events have imposed significant impacts on the Earth's environment, with far-reaching consequences for terrestrial carbon stock and ecosystem functioning [1–4], raising questions about the causes driving global wildfire dynamics. However, understanding the underlying reasons for these changes is a complex task. Wildfires require the confluence of several conditions: sufficient and continuous fuel, high fuel aridity, ignition and favorable weather conditions [5]. Climate change has been widely recognized as an important driver in amplifying fire weather conditions [6], mainly by rising temperatures and declining atmospheric humidity [7]. However, the impact of climate change on wildfires can be modulated by changes in vegetation productivity and phenology through the bioclimatic interactions amongst climate, vegetation and wildfire, either exacerbating or mitigating the risks [6]. Therefore, it is crucial to gain a better understanding of how vegetation dynamics drive wildfires to improve our capability to predict fire's future [8–10].

**Figure 1.**
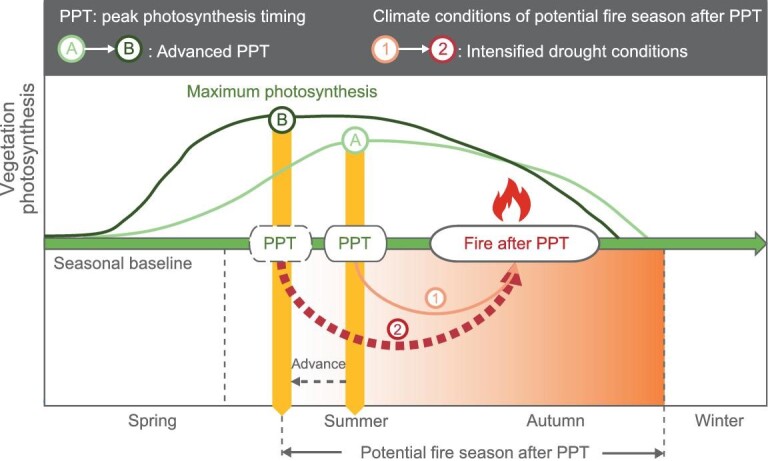
Conceptual model of wildfire response to advanced peak photosynthesis timing (PPT), bridged by the intensified drought conditions during the potential fire season after PPT. The PPT in our study was estimated by the long-term satellite-based SIF observations. In response to changes in various biological and climatic factors, PPT has advanced in many ecosystems during the past few decades [13,15], which is shown as the switch from state A to B (green). The changes in vegetation phenology can in turn feedback to the climate system by regulating the exchanges of water and energy between land and the atmosphere [16], causing intensified drought conditions during the potential fire season after PPT [climate conditions switch from state 1 to 2 (red)], and further contributing to the wildfire activity after PPT. The potential fire season after PPT is identified as being from the month of PPT to the end of autumn (November).

Peak photosynthesis timing (PPT), indicating the timing of the vigor of vegetation photosynthetic activity reaching its maximum, plays an important role in shaping terrestrial ecosystem productivity [11]. Both ground- and satellite-based observations have evidenced an advanced PPT in northern ecosystems, in response to changes in various biological rhythms and climatic factors [12–14] (Fig. 1, vegetation state A versus B). Yet, previous studies on long-term changes in PPT have been limited to specific locales or regions, except for a global analysis of phenology showing an advanced PPT in northern ecosystems and some savannas confined to the southern hemisphere [15].

Importantly, changes in vegetation phenology can modulate seasonal cycles of energy and water fluxes and landscape properties through biophysical and biogeochemical processes, thereby in turn feeding back to climate at the local-to-regional and even global scales [16,17]. For example, an earlier spring onset of vegetation growth may reduce surface temperature in spring through enhancing evaporative cooling [18], but exacerbate summer soil drying due to a large increase in evaporative water loss [19]. This prolonged soil water depletion may subsequently invert the spring cooling effect and cause more severe and longer droughts and heat waves in summer and autumn [19,20]. The advanced leaf-out additionally enhances annual surface warming through its feedback on atmospheric water vapor, snow albedo and solar radiation [21]. These studies are mainly focused on spring phenology in the northern hemisphere mid-high latitudes. However, the climatic influence of the changes in PPT and its implication on global wildfires (Fig. 1, climate state 1 versus 2) remain elusive.

Given the feedback of vegetation phenology to the climate system, we hypothesize that vegetation summer phenology (i.e. PPT) could potentially contribute to the spatial extent of subsequent wildfires (Fig. 1). To explore this, we conducted a global comprehensive analysis using various data sets and models. We analyzed long-term series (2001–2018) of regional fire perimeters (Fig. S1 in the online supplementary data), satellite-derived global burned area (BA) (Fig. S2), maximum photosynthesis and its timing from multiple satellite solar-induced chlorophyll fluorescence (SIF) data (Figs S3 and S4), and climatic variables (Table S1). We also employed an Earth System model to investigate the feedback loops between advanced PPT and the subsequent potential fire-season drought conditions globally. Furthermore, we examined the effects of PPT on BAs in an ensemble of state-of-the-art fire-vegetation models participating in the Fire Model Intercomparison Project (FireMIP; Table S2), using random forest and explainable machine-learning methods. Integrating diverse data, models and methods enables us to understand the feedbacks of vegetation phenology to climate conditions, and subsequent wildfire activity.

## RESULTS

### Global trend in PPT and its control of burned area

**Figure 2.**
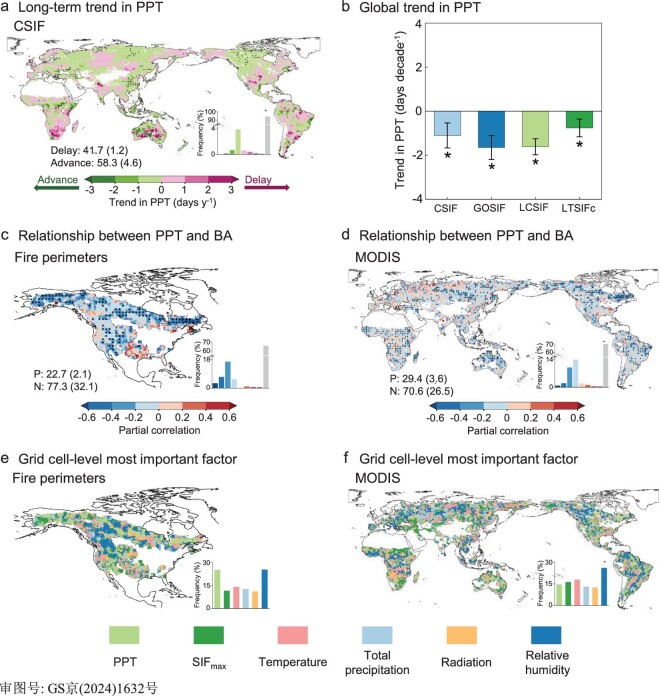
Global long-term trend in PPT and its control of burned area (BA). (a and b) Global patterns of long-term trend in PPT over 2002–2018 derived from multiple SIF products (CSIF, GOSIF, LCSIF and LT_SIFc). (c and d) Partial correlations between CSIF-derived PPT and BA based on regional fire perimeters and MODIS global BA products. (e and f) Grid cell-level most important factor in controlling variations of BA based on the partial correlations between each factor and BA. Black dots indicate the regions with significant trends or correlations (*P*-value < 0.05). Labels in (a) indicate the percentage of areas showing delayed and advanced PPT. Bar and error bar in (b) indicate decadal trend of global PPT and corresponding 95% confidence interval, respectively. The asterisk denotes *P*-value < 0.05. P and N in (c and d) denote the percentage of positive and negative correlations, respectively. The partial correlation was based on the year-to-year variations of PPT, BA and other driving factors (Methods).

The long-term vegetation photosynthesis observations from contiguous SIF (CSIF) showed that PPT was overall earlier from 2002 to 2018 across the globe, with 58.3% of areas exhibiting advanced trends, mainly in the mid-high latitude of the northern hemisphere and some savannas (Fig. 2a). Globally, PPT has advanced by 1.10 ± 0.57 days per decade (*P*-value < 0.05; Fig. 2b), with maximal advance occurring in the tropical and cold regions (−4.38 ± 1.70 and −1.33 ± 0.55 days decade^−1^, respectively, both *P*-values < 0.05; Fig. S5b–e). Across the biomes, tropical and subtropical forests, boreal forests and tundra showed more pronounced advanced trends in PPT (Fig. S5f–p). In contrast, the remaining 41.7% of areas showed delayed PPT, mostly in eastern North America, western Siberia, South Asia and southern Africa (Fig. 2a). The overall global earlier PPT was also found in the other three SIF products (GOSIF, LCSIF and LT_SIFc; Fig. 2b).

Relating year-to-year variations of BA with year-to-year variations of PPT (Methods), we found significant effects of PPT on the subsequent BA (Fig. 2c and d). Across broad swathes of Canada and the USA (32.1%), an earlier PPT led to significant (*P*-value < 0.05) expansion of BA, as determined by fire perimeters from the National Burned Area Composite (NBAC) and Monitoring Trends in Burn Severity (MTBS) (Methods), whereas a decreasing effect was only found for 2.1% of pixels (Fig. 2c). Regarding BA from the Moderate Resolution Imaging Spectroradiometer (MODIS) at global scale, we also observed a predominantly negative correlation with PPT, with 26.5% of global areas showing a significant increase in BA with earlier PPT, whereas the opposite was found for only 3.6% of pixels (Fig. 2d). Consistent results were obtained from analyses of the other three SIF products (Fig. S6). In addition to the extent of fire, we also found that earlier PPT might amplify global fire intensity, characterized by fire radiative power (FRP), with much larger areas showing negative correlations between PPT and FRP (Fig. S7).

Relative to other biological (maximum photosynthesis, noted as SIF_max_) and environmental (temperature, precipitation, solar radiation and relative humidity) cues, PPT played a key role in Canada and the USA when using fire perimeters data, with 25.3% of areas showing PPT as being the most important factor in controlling the subsequent BA, comparable to relative humidity (25.4%; Fig. 2e). Across the globe and in different ecoregions, when using MODIS BA data (Fig. 2f and Fig. S8), climate dominated the expansion of BA. Relative humidity and temperature were the most important factors in ∼47.8% of the globe (30.0% and 17.8%, respectively; Fig. 2f). PPT had a secondary effect on the subsequent expansion of BA (14.5% of the globe; Fig. 2f).

Additionally, we compared the relative importance of PPT and biomass available for burning in controlling variations in BA. Biomass available for burning is represented by SIF_max_ and pre-PPT accumulated net primary productivity (NPP) (Methods). When using NPP instead of SIF_max_, we consistently found that PPT negatively correlated with subsequent BA, whereas NPP showed a positive correlation (Fig. S9). SIF_max_ was the most important factor in ∼16.2% of global areas, slightly more than PPT at 14.5% (Fig. 2f). These comparable fractions were a result of different dominance between them across different climate zones and ecoregions. PPT was more important in tropical regions and boreal forests, whereas SIF_max_ dominated in arid regions, Mediterranean ecoregions and non-forest areas (Fig. S10a and b). These findings were also supported by using NPP as a proxy for biomass available for burning (Fig. S10c and d). In summary, although climate change remains the primary factor in large parts of the world, PPT predominantly shows a negative correlation with subsequent BA on a global scale, suggesting that earlier PPT may contribute to the subsequent expansion of fires.

### Causal mechanisms for the linkage between PPT and burned area

**Figure 3.**
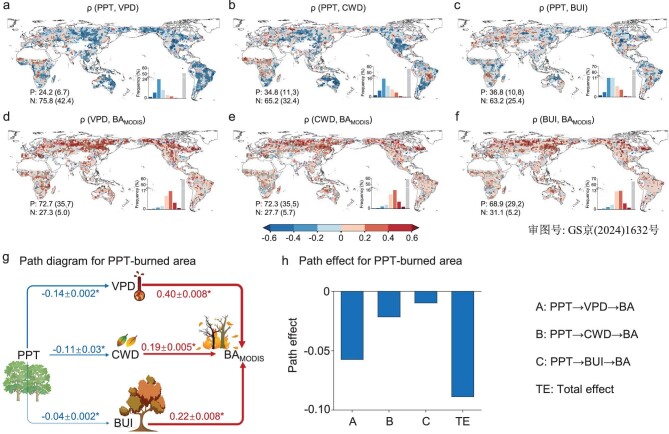
Causality networks for the potential mechanisms underlying the linkage between PPT and BA. Global patterns of partial correlations: PPT versus subsequent (a) VPD, (b) CWD and (c) BUI, and factors versus (d–f) BA. Black dots indicate the regions with significant partial correlations (*P*-value < 0.05). (g) Path diagram and (h) path effect for the linkage between PPT and BA using structural equation models (SEMs) for the globe. The numbers in the path diagram represent the global means and 95% confidence intervals of standardized path coefficients. The asterisks indicate that the path coefficients are significant (*P*-value < 0.05) and the colors (red and blue arrows represent positive and negative effects, respectively) and the widths of the arrows represent the signs and magnitudes of the path coefficients, respectively.

How does an earlier PPT contribute to the subsequent escalation of wildfires? Our analyses provided additional insights into the relationship between PPT and subsequent BA, highlighting the potential mediating role of multiple factors during the potential fire season after PPT (Fig. 1), including the vapor pressure deficit (VPD, indicating atmospheric aridity and fire weather), climatic water deficit (CWD, indicating plant water stress) and build up index (BUI, indicating potential fuel availability) (Fig. 3). We calculated the partial correlations between PPT and these factors by excluding the effects of climatic and biological conditions after PPT (Methods). We found that PPT exhibited an overall negative correlation with subsequent VPD, with 42.4% of pixels showing significant (*P*-value < 0.05) increases in VPD with earlier PPT. In contrast, the opposite was found for only 6.7% of pixels (Fig. 3a). Similar patterns were observed for the CWD and BUI, with the majority of global areas showing increases in CWD and BUI with earlier PPT (Fig. 3b and c). Considering the impacts of VPD, CWD and BUI on BA (Fig. 3d–f), these variables may act as mediators between PPT and subsequent BA. These climate feedbacks arising from earlier PPT were also supported by the other SIF (Fig. S11) and fire perimeter (Fig. S12) data.

To better understand the causal connections between these variables, we applied a structural equation model (SEM), establishing three pathways linking PPT and subsequent BA: PPT→VPD→BA, PPT→CWD→BA and PPT→BUI→BA (Fig. S13). Consistent with the partial correlation analyses (Fig. 2d), the SEM showed that earlier PPT led to increased BA at a global scale (Fig. 3g and h). The most pronounced pathway affecting the BA was the VPD effect (‘PPT→VPD→BA’ path; Fig. 3h), because of the largest standardized path coefficients for the VPD response to the change in PPT (−0.14) and the control of VPD on the BA (0.40) at a global scale (Fig. 3g and Fig. S14). For different climate zones and biomes, an earlier PPT can increase VPD, CWD and BUI and further expand BAs (Fig. S15). These potential mechanisms were also supported by other photosynthesis and BA data (Fig. S16).

It is noteworthy that the antecedent climate conditions before PPT may have carryover effects on those after due to the lagged dependencies of climate variables, i.e. climate memory effect. This temporal autocorrelation of climate conditions may confound the identification of the climate feedback arising from advanced PPT. Therefore, we further eliminated the effects of pre-PPT climate variations (i.e. temperature and precipitation) in the analysis of partial correlation. Consistently, we still found increased VPD, CWD and BUI during the post-PPT fire season with an earlier PPT (Fig. S17a–c). Moreover, we further employed random forest to eliminate the information of pre-PPT temperature and precipitation from the original post-PPT VPD, CWD and BUI and developed an SEM as in Fig. S13 (Methods). The consistent results (Fig. S17d and e) confirm that intensified drought conditions can stem from the climate feedback of advanced PPT, rather than being artificially caused by climate memory effect. Overall, these analyses provide solid evidence that an earlier PPT enhances subsequent wildfire activity, potentially by increasing the VPD, CWD and BUI that follow.

### CESM simulations of PPT feedback to climate

Besides data mining, numerical modeling can provide further insights into the climate feedback resulting from advanced summer phenology at a global scale, shedding light on how potential fire-season weather conditions respond to an earlier PPT. We performed two 100-year simulations using the Community Earth System Model 2.2 (CESM) with coupled land and atmosphere components. In short, we started with a ‘climatological phenology’ experiment that applied default prescribed satellite vegetation phenology (monthly climatological leaf area index and stem area index) to the model. By shifting the global vegetation growing phase in summer (i.e. June, July and August for the northern hemisphere and December, January and February for the southern hemisphere) earlier by 10 days, we carried out an ‘advanced phenology’ experiment that represented earlier vegetation phenology in the model. As the PPTs of most vegetated lands occur in summer (Fig. S18), the simulated differences between these two experiments largely represent the influence of an earlier PPT (see Methods for details).

**Figure 4.**
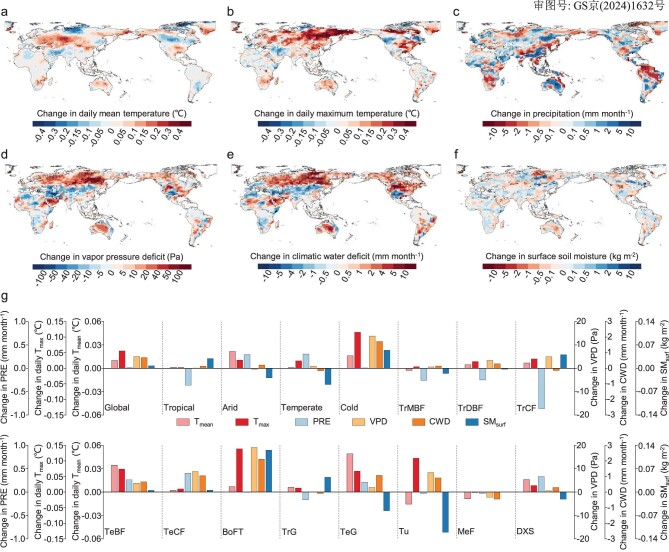
Earth system simulations of the global effects of advanced summer vegetation phenology on climate parameters. Changes in daily mean and daily maximum (a and b) surface air temperature, (c) precipitation, (d) VPD, (e) CWD and (f) surface soil moisture content at a depth of 0–10 cm in summer and autumn induced by advanced summer vegetation phenology. The black dots mark the regions with a statistically significant change between sensitivity and control simulations (Student's t-test, *P*-value < 0.05). (g) Averaged changes in 6 climate parameters induced by advanced summer phenology for the globe, 4 climate zones, and 11 biomes. Climate zones were derived from the Köppen-Geiger climate classification map (Fig. S19). Biomes were derived from the Terrestrial Ecoregions of the World (Fig. S20).

Our simulations showed that a 10-day earlier PPT led to enhanced summer-autumn warming, increasing global summer-autumn daily mean temperature and daily maximum temperature by 0.01°C and 0.06°C, respectively (Fig. 4a, b and g), indicating an increased probability of heatwaves in summer and autumn. This earlier-PPT-induced warming was more remarkable in most northern hemisphere mid-high latitudes and some arid regions in southern Africa and Australia (Fig. 4a and b), contributing to higher VPD and CWD there (Fig. 4d, e and g). The patterns of the simulated VPD and CWD changes due to earlier PPT generally align with those of observed relationships between PPT and VPD/CWD (Fig. 4d and e vs. Fig. 3a and b).

Earlier summer phenology also modified precipitation patterns and soil moisture. Specifically, in broad swathes of tropics, advanced summertime vegetation activity resulted in reduced rainfall during summer and autumn (Fig. 4c and g), which combines with warmer air and leads to increased tropical VPD and CWD (Fig. 4g). Additionally, surface soil moisture was reduced in tundra ecosystems and arid and temperate non-forests due to increased evaporative demand induced by higher temperatures and earlier enhancement of vegetation activity, despite an increase in rainfall (Fig. 4f and g). In such regions with strong soil moisture-climate coupling, the reduced soil moisture will in turn feedback to the atmosphere and result in more severe hot and dry conditions by suppressing evaporative cooling and increasing sensible heat (Fig. 4g). Therefore, Earth System modeling confirms that advanced PPT tends to exacerbate subsequent drought conditions through biophysical feedback processes, creating more favorable conditions for wildfires.

### Diagnosis of global fire sensitivity to PPT in FireMIP models

**Figure 5.**
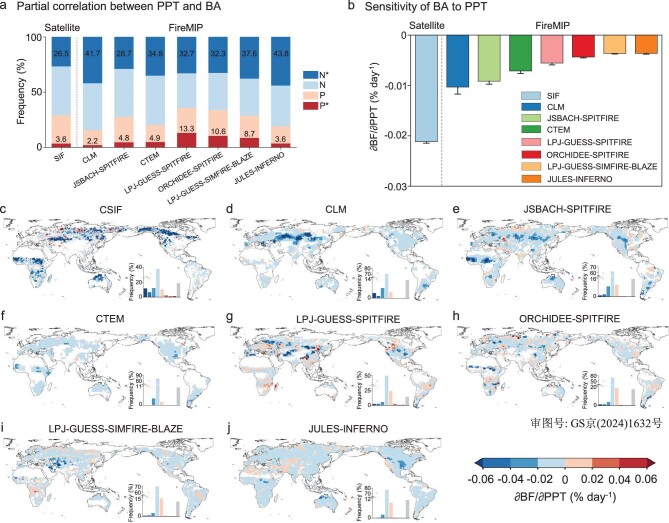
Comparisons of the effects of PPT on BA from satellite observations and FireMIP fire-vegetation models. Comparisons in terms of (a) partial correlation and (b) sensitivity. Labels in (a) indicate the percentages of significantly negative and positive correlations (*P*-value < 0.05). Bars and error bars in (b) indicate the global area-weighted mean and 95% confidence interval of sensitivity, respectively. (c–j) Global patterns of sensitivity of BA to PPT for (c) observation-based CSIF, and seven FireMIP models including (d) CLM, (e) JSBACH-SPITFIRE, (f) CTEM, (g) LPJ-GUESS-SPITFIRE, (h) ORCHIDEE-SPITFIRE, (i) LPJ-GUESS-SIMFIRE-BLAZE and (j) JULES-INFERNO derived from random forest and SHAP methods. BA in sensitivity analysis was represented by burned fraction (BF, %). The gray area indicates that the sensitivity is not statistically significant (*P*-value > 0.05).

The impact of PPT on subsequent BAs derived from satellite-based observations provides a benchmark to assess whether state-of-the-art fire-vegetation models can reproduce this relationship. Therefore, we used seven models participating in the FireMIP that simulate seasonal variations in vegetation photosynthesis (represented by gross primary productivity, GPP) and wildfire activity (represented by BA). We found that the FireMIP models were able to replicate the correlation between PPT and BA at a global scale, showing a predominantly negative relationship that aligned with that from satellite observations (Fig. 5a). However, further sensitivity analysis based on random forest and explainable machine-learning (Shapley Additive Explanations, SHAP) methods revealed that FireMIP models could not reproduce the magnitude of this effect. On average, the models underestimated the global sensitivity of BA to PPT by 240% when compared with satellite observations (−0.0062% day^−1^ for model average vs. −0.021% day^−1^ for CSIF; Fig. 5b). The underestimations were mainly shown in the arid and high-latitude cold regions (Fig. 5c–j). The Community Land Model (CLM), which serves as the land model for the CESM, performed comparatively better than the other FireMIP models in replicating the observed global sensitivity of BA to PPT (−0.010% day^−1^), but still underestimated the sensitivity by 105% (Fig. 5b–d). Additionally, the global sensitivities estimated from other SIF observations ranged from −0.024 to −0.034% day^−1^, consistently higher than those from FireMIP models (Fig. S21).

## DISCUSSION

Climate change has been widely recognized as an important determinant driving the increased extreme fire weather conditions [7,22] and the recent surge of extreme wildfire events in some ecosystems [23,24]. Our results support that climate is the primary factor influencing variations in BA (Fig. 2f). However, the bioclimatic interactions between climate and vegetation make it more complex. In the long term, climate affects vegetation type and distribution, and thus affects fire regimes [25]. Additionally, climate change and variability also modulate vegetation growth and moisture conditions at seasonal and interannual scales [26]. Meanwhile, the seasonal growth of vegetation and some critical timings will, in turn, feedback to the climate system and landscape [16]. Therefore, we argue that the influence of vegetation seasonal growth and phenology on fire dynamics cannot be overlooked. Previous studies have proved that spring vegetation phenology and the seasonal variability in the normalized difference vegetation index (NDVI) can contribute to wildfire activity at a regional scale [27–29]. Our study provides a novel perspective for understanding the bioclimatic interactions amongst climate, vegetation and wildfire by demonstrating the biophysical feedbacks from summer vegetation phenology (i.e. PPT) to subsequent climate and wildfire activity at a global scale.

Our results highlight the potential amplifying effects of earlier PPT on subsequent BAs at a global scale, and were supported by various sources of satellite observations and different methodologies. PPT has a secondary effect on fire activity, following climate change (Fig. 2f). Advancing PPT corresponds with increasing SIF_max_ across large areas of the globe (Fig. S4), indicating a greater availability of live biomass for combustion (Fig. S22). Thus, we also compared the relative importance of PPT and available biomass. Their different dominance across different ecoregions results in their comparable importance in controlling fire activity on a global scale (Fig. S10). PPT is more important in tropics and boreal forests, where fire is mainly limited by drought conditions [30], partly related to climate feedbacks from earlier PPT. Available biomass is more important in arid regions and non-forest areas, where fire is dominated by the amount of fuel [30].

In some deciduous ecosystems and grasslands, the process of vegetation senescence begins after PPT, transitioning biomass from alive to dead. The accumulation of dead fine fuel can lead to increases in fire extent and intensity [31–33], because it possesses much lower moisture content than live fuel, making it more susceptible to dry weather conditions, and more flammable [34]. Additionally, the fuel gets drier throughout dry conditions from summer to autumn, which may be further enhanced by heat and water deficit induced by the biophysical feedback of vegetation phenology (Figs 3 and 4). These processes may increase fire activity.

For the fire ignition source, we have excluded cropland fires to partly reduce the impact from human fire use, as croplands are human-dominated ecosystems (Methods). However, we do not explicitly distinguish all the natural or anthropogenic causes of BA. Human activity can alter fire dynamics though ignition, active fire suppression, and manipulations of the timing and fuel conditions of fires [35,36], potentially confounding the identification of PPT–fire interactions. Differentiating between anthropogenic and natural fires is essential to improving our understanding of global fire dynamics, but it remains a challenging task [37]. Considering that human activities often lead to land-use and land-cover changes (LULCC), we excluded the burned pixels where LULCC occurred during the study period. To some extent, this can mitigate the influence of human-caused fires (Methods). Our main results remained unchanged (Fig. S23). In addition, we used the low-human-impact area (LIA) and intact forest to distinguish between nature- and human-dominated areas. We found that PPT–fire interactions were stronger in natural landscapes (Figs S24 and S25), consistent with a recent study suggesting that lower human impact coincides with a stronger climate–fire relationship [37].

Moreover, we investigated the ability of FireMIP models to replicate the observed effects of PPT on BA. FireMIP models have been shown to adequately represent the seasonal peak timing in GPP and the seasonality of BA [8], explaining why they were able to replicate the direction of relationship between PPT and BA. However, our results from the random forest and SHAP also reveal that the FireMIP models do not capture the magnitude of this effect, underestimating the global sensitivity of BA to PPT by up to 240% compared to satellite observations. The discrepancy may arise from the omission of vegetation feedbacks to the climate system in offline simulations, and the failure to adequately represent pre-season fuel build-up and its subsequent effect on BAs in the models [8,38]. Therefore, detailed representations of changes in vegetation productivity and phenology, and fire-vegetation-climate feedbacks, could improve the predictive capacity of fire models.

Using empirical and mechanistic models, we attempted to understand the global connection between PPT and wildfires, considering both biophysical and biogeochemical processes. An earlier PPT results in higher BUI in the subsequent summer and autumn, meaning a larger potential amount of fuel available for combustion [39]. In addition, Earth System modeling strengthens the causal linkage between PPT and subsequent BAs, bridged by amplified drought conditions. The biophysical feedback from earlier PPT exacerbates atmospheric and fuel aridity. The intensified drought conditions in different regions can be attributed to different processes. At the global scale and for the boreal forests, increased VPD and CWD mainly results from increased mean and extreme temperatures. In tropical forests and savannas, decreased precipitation induced by earlier summer phenology is more pronounced. For tundra, arid regions and some temperate grasslands, higher temperatures and reduced soil moisture play the dominant role, due to the strong soil moisture-climate coupling in these areas [40]. However, future studies still need to disentangle the complex biophysical and physiological processes linking vegetation phenology, climate conditions and fire activity by means of observations and Earth System models, ultimately benefiting the improvement of fire dynamics modeling.

## CONCLUSIONS

This study investigated the influence of PPT on the subsequent BA at a global scale, from the perspective of the biophysical climate feedbacks arising from vegetation phenology dynamics. Our results revealed that earlier PPT could potentially expand the subsequent BA globally. This fire escalation is likely driven by the interplay of multiple factors, including increased atmospheric aridity, plant water stress and accumulated fuel availability associated with the climate feedbacks arising from earlier PPT. Current fire-vegetation models notably underestimated the effects of PPT on BA.

Although climate change remains the primary driver of global fire dynamics, the impact of vegetation growth and phenology cannot be overlooked. Variations in the plant growth phase have the potential to alter the temporal distribution of fuel sources, and the biophysical feedback resulting from these changes can affect regional temperature, precipitation and soil moisture after PPT. Given the continued PPT advancement due to climate change and the resultant upward pressure placed on wildfires, our findings highlight an urgent need to incorporate the bioclimatic effects of vegetation phenology change on wildfires in fire modeling and climate change adaptation and mitigation.

## DATA AND METHODS

We mainly used multiple data sets (Tables S1 and S2), including four SIF products, a MODIS global BA product, regional fire perimeters from NBAC and MTBS, climatic data, and the outputs of vegetation productivity and BA from fire-vegetation models participating in the FireMIP. We combined multiple statistical techniques (partial correlation, SEM, random forest and the SHAP methods) and an Earth System model to elucidate the relationship between PPT and BA, and the underlying mechanisms between them. The analyses were mainly designed to detect the biophysical feedbacks from shift in PPT to the subsequent climate conditions, thus we focused on the period of potential fire season after PPT, identified as from the month of PPT to the end of autumn (Fig. 1). Finally, we diagnosed the capability of current fire-enabled dynamic global vegetation models to reproduce the observed relationship between PPT and BA. See Supplementary Methods for extended data and methods.

## Supplementary Material

nwae292_Supplemental_File
