# Supplementary material for: Earlier peak photosynthesis timing potentially escalates global wildfires
Source: Natl Sci Rev. 2024 Aug 22;11(9):nwae292. doi: 10.1093/nsr/nwae292 (PMC11418649; doi:10.1093/nsr/nwae292)
Supplement: nwae292_Supplemental_File [file nwae292_supplemental_file.pdf]

Supplementary Information

Earlier peak photosynthesis timing potentially escalates global wildfires

Gengke Lai[1,2,†], Jialing Li[1,2,†], Jun Wang[3,†], Chaoyang Wu[3,*], Yongguang Zhang[1,2,4,8,*], Constantin M. Zohner[5], Josep Peñuelas[6,7,8], Quansheng Ge[3,*]

[1]International Institute for Earth System Sciences, Jiangsu Center for Collaborative Innovation in Geographical Information Resource Development and Application, Nanjing University, Nanjing 210023, China;

[2]Jiangsu Provincial Key Laboratory of Geographic Information Science and Technology, Key Laboratory for Land Satellite Remote Sensing Applications of Ministry of Natural Resources, School of Geography and Ocean Science, Nanjing University, Nanjing 210023, China;

[3]The Key Laboratory of Land Surface Pattern and Simulation, Institute of Geographic Sciences and Natural Resources Research, Chinese Academy of Sciences, Beijing 100101, China;

[4]Huangshan National Park Ecosystem Field Scientific Observation and Research Station of the Ministry of Education, Nanjing 210023, China;

[5]Department of Environmental Systems Science, Institute of Integrative Biology, ETH Zurich, Zurich 8092, Switzerland;

[6]CSIC, Global Ecology Unit CREAF-CSIC-UAB, Barcelona 08193, Spain;

[7]CREAF, Cerdanyola del Vallès, Barcelona 08193, Spain;

[8]Jiangsu International Joint Carbon Neutrality Laboratory, Nanjing University, Nanjing 210023, China.

***Corresponding authors.** E-mails: wucy@igsnrr.ac.cn; ygz@nju.edu.cn; geqs@igsnrr.ac.cn

[†]Equally contributed to this work.

Supplementary Methods

Satellite-based global vegetation photosynthesis

The recent emergence of solar-induced chlorophyll fluorescence (SIF) serves as a direct indicator of vegetation photosynthesis activity [1-3] because it is a by-product of photosynthesis [4]. Recent advances in the retrieval of SIF from satellite observation allow for monitoring the seasonal variations of vegetation photosynthesis from regional to global scales [5, 6]. Thus, we used SIF as a metric to estimate vegetation photosynthesis phenology at different spatiotemporal scales [7-9]. To explore the robust trends in peak photosynthesis timing (PPT) and its impacts on subsequent wildfire activity, we employed three global reconstructed SIF products from 2001–2018 with diverse spatiotemporal resolutions and methodologies of processing and retrieval: (i) the clear-sky SIF from the contiguous SIF (CSIF) dataset with 0.05 °spatial- and 4-day temporal-resolution [10]; (ii) the global ‘OCO-2’ SIF (GOSIF) dataset with 0.05° spatial- and 8-day temporal-resolutions [11]; (iii) and the global long-term contiguous SIF (LCSIF) dataset with a 0.05 °spatial resolution and a bi-monthly temporal resolution [12]. The three reconstructed datasets are not completely independent SIF products, because all of them were generated from the Orbiting Carbon Observatory-2 (OCO-2) SIF and MODIS reflectance observations and/or meteorological reanalysis data by using machine learning or neural network. Therefore, we further utilized a monthly temporally corrected long-term satellite SIF (LT_SIFc) dataset from 2001 to 2018 with a spatial resolution of 0.05 °to track the seasonal variations of photosynthesis. This dataset integrates three satellite-based SIF products, including Global Ozone Monitoring Experiment (GOME), Scanning Imaging Absorption Spectrometer for Atmospheric Chartography (SCIAMACHY), and GOME-2, correcting the temporal inconsistency of these three products [13].

The order of magnitude of current PPT trends are days, so we selected CSIF with the highest temporal resolution (4 days) as the primary product, with other three SIF (GOSIF, LCSIF, and LT_SIFc) products being used for validation.

Extraction of global peak photosynthesis timing

Before fitting the photosynthesis phenology curves, we first identified the start and end day of year (DOY) for the whole year: From January 1st to December 31st for the Northern Hemisphere, and July 1st in the previous year and June 30th in current year for the Southern Hemisphere.

We used the method of combining Savitzky-Golay (SG) filter and cubic spline [14] to extract PPT and corresponding maximum photosynthesis. This method is flexible to fit a wide range of seasonal variations of vegetation [15], and is effective in capturing the peak photosynthesis timing at large scale [8, 14]. For each pixel per year, the first step was using the SG filter to smooth the SIF time series. The smoothing window size was set as 7 for CSIF, GOSIF, and LCSIF to get the

resultant value positioned at the center of the window. However, given the coarse monthly resolution of the LT_SIFc data, the window size was set as 5 for it. Then, the day of the year with maximum SIF value (SIF_{max}) was considered as PPT based on the daily SIF reconstructed by cubic spline. Accordingly, we estimated global yearly PPT and maximum photosynthesis from 2002 to 2018 at the native grids of each product. Then, the estimated PPT and maximum photosynthesis were upscaled to 0.25 °using the nearest-neighbor and bilinear interpolation methods, respectively. In addition, to mitigate the influence of abnormally high values in SIF_{max}, we used the 95th quantile to remove outliers.

Despite being widely used in fire research, the spatial scale of 0.25 °may result in the mixed pixel problem that contain different vegetation types and confound the detection of PPT trend and its impact on subsequent wildfires. Therefore, we performed an additional analysis at a finer spatial resolution (0.05 °). Consistently, we found an overall advanced PPT at a global scale (Fig. S26a), and a predominantly negative correlation between PPT and subsequent burned area (Fig. S26b).

We calculated long-term trends in PPT at the grid level using the Theil-Sen slope estimator in combination with the Mann-Kendall test. In addition, to quantify the PPT trends for the globe, 4 climate zones, and 11 biomes, we computed the regional median PPT values for each year and used 5-year moving average approach to remove high-frequency variability and potential outliers [16], and estimated PPT trends and corresponding 95% confidence intervals via linear regression. All SIF observations confirm an overall advancement in PPT at a global scale, with global PPT trends from -0.76 ±0.40 days decade^{-1} for LT_SIFc to -1.65 ±0.54 days decade^{-1} for GOSIF (Fig. 2b). The trends of PPT in northern ecosystems (>30 °N) were from -0.90 ±0.44 days decade^{-1} for LCSIF to -2.32 ±0.80 days decade^{-1} for GOSIF (Fig. S27), which were consistent with previous studies that reported the trend of PPT in northern ecosystems at -0.83 ~ -1.66 days per decade [8, 16, 17]. The slight discrepancy may arise from the differences in study period, retrieved methodology and data sources. Generally, it demonstrates that our method used to calculate PPT is feasible and reasonable.

Fire perimeters

We utilized fire perimeter products from Canada (National Burned Area Composite, NBAC) and the US (Monitoring Trends in Burn Severity, MTBS) to extract information on burned area (BA) occurring after PPT for the period of 2002–2018. NBAC, developed by the Canada Centre for Mapping and Earth Observation and the Canadian Forest Service, provides yearly burned areas on a national scale since 1986. This dataset is derived from three burned area data sources, including the Canadian National Fire Database (CNFDB) agency data, finer-resolution burned area product from Landsat imagery, and coarse-resolution burned area product from SPOT VEGETATION/Proba-V imagery [18]. NBAC provides two layers of information on fire start

date: one is based on the first detected hotspot (SDATE) and the other is reported by the fire management agency (AFSDATE). Given no unified burned area mapping method and data source for all fire management agencies across Canada, which may create uncertainties in burned area estimates from AFSDATE [18], we took SDATE as the preferred information, and AFSDATE as the potential source if SDATE is not available. Based on this criterion, the yearly gridded burned area occurring after PPT was derived by three steps: (i) the fire perimeters were initially rasterized at a spatial resolution of 0.00025°, ensuring good correspondence between fire pixels and perimeters; (ii) the values of SDATE and AFSDATE were allocated to each finer-resolution pixel, respectively; (iii) the finer-resolution pixels with fires occurring after PPT were identified based on the above criterion, and then used to calculate total burned area (expressed as burned fraction with a unit of %) in the 0.25° grid cell.

MTBS, developed by the U.S. Geological Survey Center for Earth Resources Observation and Science (EROS) and the USDA Forest Service Geospatial Technology and Applications Center (GTAC), provides national burn severity and extent of large fires since 1984 (https://www.mtbs.gov/). We used the national burned area boundaries dataset (i.e. fire perimeters) from 2002 to 2018. Given the much smaller extent of Hawaii and Puerto Rico, we only considered the continental U.S. and Alaska. This dataset is generated by primarily leveraging Landsat imagery at a 30-m resolution, and Sentinel-2 as a complemental data source since 2015 when there were no available high-quality Landsat observations [19]. The dataset provides information on fire incident type (wildfire or prescribed fire) and ignition date. We only considered wildfires, and followed the same processing steps as NBAC to calculate yearly gridded burned areas that occur after PPT.

Accordingly, we obtained yearly gridded burned areas occurring after PPT with a 0.25° resolution in Canada and the US from 2002 to 2018. Note that though NBAC and MTBS datasets have been widely used in assessing fire dynamics and their attributions [20-23], it should be cautious when using these national-scale fire perimeters data. Because they are generated through completely different approaches and from different data sources, and even within one product some discrepancies could create uncertainties in the estimated burned area boundaries [18, 19]. For example, NBAC is derived from three data sources, and there is no unified method and data source for all fire management agency data [18]. Thus, we chose the detected hotspot as the preferred data source to partly mitigate the uncertainties from fire management agency data. Moreover, in the case of MTBS, the minimum burned area mapping units are different in the western and eastern U.S., being 1000 acres and 500 acres, respectively [19]. Therefore, we opted to rasterize these different fire perimeter data and conduct our time-series analyses at the grid level. This approach can mitigate potential uncertainties arising from combining these data with large

discrepancies. It should be noted that the estimate of the impact of PPT advancement on the following burned area is conservative when using the MTBS because of its omission of small fires.

MODIS global burned area

We also used the MODIS global monthly burned area product (MCD64A1 V6) with 500-m resolution [24] over 2001–2018 to examine the impacts of PPT on subsequent burned areas at the global scale. The product provides information on burn date (expressed as DOY). We calculated the gridded burned area after PPT with a 0.25 °resolution for each year through the following steps: (i) the start and end days for each year were identified, aligning with the global PPT calculation; (ii) any burned pixels located in croplands and non-vegetated areas were masked out based on the IGBP classification scheme from MCD12Q1 V6 land cover product; (iii) the 500-m pixels were allocated into the 0.25 °grid cell, with total burned areas occurring after PPT calculated. Note that the pixels repeatedly marked as burned in one year were only recorded once in the burned area calculation.

VPD, CWD, and BUI

To elucidate the mechanistic linkages between PPT and subsequent wildfires, we considered three variables as the mediators: vapor pressure deficit (VPD), representing atmospheric aridity and fire weather [25]; climatic water deficit (CWD), indicating plant water stress [26]; and built up index (BUI), reflecting the total amount of fuel available for combustion [27]. Monthly VPD was calculated based on air temperature (T) and dewpoint temperature (Td) at 2 m above the surface following the ref. [28], derived from ERA5-Land monthly data provided at a 0.1 ° resolution [29]. Monthly CWD, calculated as the difference between potential and actual evapotranspiration, was obtained from the TerraClimate dataset with a 1/24 °resolution [30]. BUI was derived from the fire danger indices historical data produced by the Copernicus Emergency Management Service for the European Forest Fire Information System (EFFIS). This dataset provides daily 0.25 °fire danger indices for three different models developed in Canada, the US, and Australia [27]. Note that BUI, a key component of the Fire Weather Index System, represents the potential availability of accumulated fuel, thereby also serving as an effective drought indicator. Increased BUI typically signifies enhanced drought conditions [31]. Daily BUI was arithmetically averaged for each month and aggregated over the fire season after PPT. These three variables were upscaled to 0.25 °using bilinear interpolation.

Other climatic and auxiliary data

To eliminate the compounding effects of other climatic factors on the post-PPT wildfires, we utilized the climatic variables derived from the ERA5-Land datasets, including 2-m T and Td, total precipitation (PRE), downwards surface solar radiation (RAD), and volumetric soil water content

at the 0-7 cm layer (representing surface soil moisture content, SM). Besides, relative humidity (RH) has been recognized as an important variable controlling wildfire dynamics by increasing fire weather [25, 32-34], which was calculated with the T and Td based on the ref. [28].

We also used fire radiative power (FRP) to represent fire intensity [35], extracted from the MCD14ML V6 active fire product for the period 2001-2018 [36]. This product provides information on hotspot detections, including coordinates, FRP, acquisition time, detection confidence, and fire type. For each year, we allocated the post-PPT vegetation fire hotspots with a detection confidence larger than 50% into 0.25° × 0.25° grid cells with the WGS84 coordinate system, and then averaged FRP to represent FRP per detection after PPT.

To test the relative importance of PPT and biomass available for burning in driving variations of burned area, we used the dataset of global simulated daily net primary productivity (NPP) from Boreal Ecosystem Productivity Simulator (BEPS) over 2001-2018 [37]. The BEPS model is driven by remotely sensed leaf area index, clumping index, and land cover type, as well as meteorological and soil data, to simulate daily NPP with a 0.072727° resolution [37]. NPP encompasses multiple processes associated with plant biomass accumulation, including carbon sequestration via gross primary productivity (GPP) and carbon loss via autotrophic respiration. Moreover, NPP has been proved to be critical for simulating fire occurrence and behavior [38, 39], and is a key output variable representing vegetation productivity, biomass accumulation and subsequent fuel development in state-of-the-art fire-enable vegetation models [40, 41]. Therefore, pre-PPT accumulated NPP can serve as a proxy for the biomass available for burning. NPP was resampled to 0.25° by using the bilinear interpolation method. Given that SIF_{max} shows a strong spatiotemporal consistency with pre-PPT accumulated NPP (Fig. S22), it can also be used as an indicator of the biomass available for burning.

Our results were explored for different climate zones (tropical, arid, temperate, and cold), derived from a Köppen-Geiger climate classification map (Fig. S19) [42], and for different biomes derived from the Terrestrial Ecoregions of the World (Fig. S20) [43]. We also used the PKU GIMMS NDVI product from combining AVHRR and MODIS observations [44], which provides quality control (QC) layer to check the quality of NDVI value. Thus, we excluded the pixels contaminated by cloud, snow and ice. The pixels with a mean annual NDVI low than 0.1 were regarded as areas with no or sparse vegetation cover, and were excluded from the analysis.

Characterization of climate conditions of potential fire season after PPT

For each year, our analyses were focused on the period from the month of PPT to November (generally from summer to autumn), representing the potential fire season after PPT that can affect the expansion of burned areas (Fig. 1). Therefore, we averaged the above-mentioned climatic variables from the month of PPT to November to characterize the climate conditions of potential

fire season, except for precipitation which was accumulated within this period. Therefore, it should be noted that when exploring the causal mechanisms linking PPT and subsequent burned area, we focused on the feedbacks of PPT on climate conditions after PPT.

Analyses

We first calculated year-to-year variations (that is, the difference between two consecutive years) of PPT, subsequent burned area, maximum photosynthesis, and the climatic variables of potential fire season after PPT (including VPD, CWD, BUI, T, PRE, RAD, SM, and RH). This treatment can disentangle the resulting signal from possible long-term dependencies on covariates [45]. For each grid cell at a 0.25° resolution, the time series of observations were constructed by combining these year-to-year variations of variables. Additionally, we only considered the grid cell in which fire changes in two consecutive years when constructing the time series. Thus, there are three situations: fire→fire; fire→no fire; no fire→fire. The analyses were performed based on these constructed time series. However, the structural equation model (SEM) was an exception. To ensure the model stability, SEM was performed based on the normalized year-to-year variations, calculated by dividing the year-to-year variations by the standard deviation. In addition, to enhance the reliability and better sample the statistical inferences by increasing the sample size, we applied a 9×9 spatial moving window (2.25°×2.25°) to represent the central grid cell during all analyses based on observations.

We applied partial correlation to investigate the relationship between PPT and subsequent burned area after removing the effects of SIF_{max}, T, PRE, RAD, and RH. The impacts of other factors on burned area were also computed to compare with PPT. When calculating the partial correlation between each factor and burned area, we excluded the effects of the remaining factors. We identified the most important factor for each grid cell based on the absolute value of partial correlation. Since PPT plays a crucial role in shaping vegetation productivity [16], as demonstrated in Fig. S4 where advancing PPT corresponds with increasing SIF_{max} across large areas of the globe, we also compared the relative importance of PPT and SIF_{max} (or pre-PPT accumulated NPP) in controlling variations in burned area. Both SIF_{max} and NPP can represent biomass available for burning (see *Other climatic and auxiliary data* for details). When comparing PPT with NPP, SIF_{max} was replaced by NPP in the partial correlation.

Considering the biophysical and biogeochemical feedbacks of vegetation phenology to the climate system and landscape, we utilized VPD, CWD, and BUI as the explanatory factors to explore the causal mechanisms linking PPT and subsequent burned area. First, we used partial correlation to elucidate the effects of PPT on these explanatory variables, and subsequently, the impacts of these variables on burned areas. Because soil moisture plays an importance role in the exchanges of water and energy between land and atmosphere [46], we excluded the effects of

temperature, total precipitation, soil moisture and maximum photosynthesis when calculating these partial correlations.

To further explore the underlying mechanisms for the linkage between PPT and subsequent burned area, the SEM was employed. Three pathways were designed: PPT→VPD→BA, PPT→CWD→BA, and PPT→BUI→BA (Fig. S13). When constructing the SEM, we also considered temperature, precipitation, maximum photosynthesis, and soil moisture as the driving factors of VPD, CWD, BUI and burned area. Note that the PPT precedes these climatic conditions (see *Characterization of climate conditions of potential fire season after PPT*), thus the path is unidirectional. That is, PPT points to VPD, CWD and BUI, representing the feedbacks of PPT to the subsequent climate, but there is no reverse that represents the climate impact on PPT. The SEM was performed at grid level based on the normalized year-to-year variations of time series, and estimated the standard path coefficient for each path to represent the magnitude and direction of the impact. Following the ref. [47], five metrics were selected to assess the goodness of fit of the model, including the Goodness-of-fit Index (GFI ≥ 0.95), Comparative Fit Index (CFI ≥ 0.90), Root Mean Square Error of Approximation (RMSEA < 0.1), Non-Normed Fit Index (NNFI ≥ 0.92), and Standardized Root Mean Square Residual (SRMR < 0.08). The model for each grid cell was deemed reliable when three out of five criteria were met [47]. The regional mean standard path coefficient was calculated for the globe, 4 climate zones, and 11 biomes, under the consideration of the goodness of fit of the model (Fig. S28) and the significance level of the path coefficient (p-value < 0.05). To further quantify the global sensitivity of burned area to PPT, we also used the random forest and explainable machine learning (Shapley Additive Explanation, SHAP) method to strengthen the robustness of the impact of PPT on subsequent burned area than traditional statistical approach (see *Random forest and SHAP* for details).

We noted that the antecedent climate conditions before PPT may persistently influence those after due to the temporal autocorrelation of climate variables [48, 49]. This effect may confound the identification of the climate feedback arising from advance PPT. Therefore, we further verified the earlier PPT-induced drought conditions in two aspects. On one hand, we further excluded the effects of pre-PPT (from March to the month of PPT) temperature and precipitation when calculating the partial correlations between PPT and VPD, CWD, and BUI. On the other hand, for each grid cell, we used random forest to model the relationships between year-to-year variations of post-PPT VPD (CWD/BUI) and year-to-year variations of pre-PPT temperature and precipitation. Then, we calculated the VPD* (CWD*/BUI*) by subtracting the effects of pre-PPT temperature and precipitation estimated by the trained model from the original factors. Finally, the estimated VPD*, CWD* and BUI* were used to develop a SEM with the same structure as Fig. S13. To increase the training sample size, we used a 9×9 spatial moving window to train the model representing the central grid cell.

Model configuration and experimental design

To strengthen the causal mechanisms regarding the climatic feedback of summer vegetation phenology, we further used the Community Earth System Model version 2.2, which is the latest version of the coupled Earth system model developed at the National Center for Atmospheric Research in collaboration with universities and other research institutions [50]. We ran the model for AMIP-type simulations with two-way coupled atmosphere (Community Atmosphere Model version 6, CAM6) and land (Community Land Model version 5, CLM5) components, and prescribed sea surface temperature and sea ice concentration of present-day climatology (1995-2005), with biogeochemistry model being inactive. The CAM6 and CLM5 use a nominal 2 ° (1.9 ° in latitude and 2.5 ° in longitude) horizontal resolution with 32 vertical levels and a model top pressure at 3.64 hPa. Satellite-observed vegetation phenology, monthly leaf area index (LAI), stem area index (SAI), and other vegetation features around the year 2000 are prescribed for each plant functional type (PFT) in CLM5. Monthly PFT LAI values were produced based on the 1-km MODIS-derived monthly grid cell average LAI [51, 52]. The SAI was calculated from the monthly PFT LAI using the method proposed by ref. [53].

To spin up the Earth System model, we first ran it for 50 simulation years starting from the prescribed initial conditions. Then we employed a consistent set of final restart files for the initial conditions in the following 100-year control and sensitivity simulations. We kept all the vegetation parameters unchanged in the control simulation, but modified the prescribed global LAI and SAI in the sensitivity simulation by shifting their summertime (June, July, and August for the Northern Hemisphere; December, January, and February for the Southern Hemisphere) growing phases earlier by 10 days. Specifically, we obtained summertime daily LAI and SAI series by linearly interpolating between monthly values and replaced the original indices with those 10 days later. Note that the values of LAI and SAI in other seasons remained unchanged. Considering that the annual maximum photosynthesis for the majority of the globe occurs in summer (Fig. S18), the simulated differences of summer-autumn climatic conditions between the sensitivity and control experiments largely represent the climatic feedback of advanced PPT.

FireMIP models

To investigate whether the state-of-the-art fire-vegetation models were able to reproduce the observed impacts of PPT on subsequent burned area, we employed the outputs of FireMIP models to compare with satellite results over 2001–2012 [54]. Note that this is the overlapping period (i.e. 2001–2012) of satellite observations and FireMIP simulations. The FireMIP aims to evaluate the advanced global fire models and promote projections of global fire dynamics and impacts on ecosystems and human societies under climate change [41]. These offline models employ empirical and process-based approaches and incorporate the impacts of climate, vegetation, and

human activities on fire dynamics, but no local land-atmosphere feedback. We utilized the seven out of nine FireMIP models (including CLM, JSBACH-SPITFIRE, LPJ-GUESS-SPITFIRE, ORCHIDEE-SPITFIRE, CTEM, JULES-INFERNO, and LPJ-GUESS-SIMFIRE-BLAZE) that output monthly gross primary productivity (GPP) and burned area (Table S2), which allows for the calculation of PPT based on the SG-cubic spline method and subsequent burned area.

We examined the abilities of these models compared with satellite observations from 2001 to 2012 in two aspects: (i) using partial correlation to qualitatively compare the correlation between PPT and subsequent burned area; ii) utilizing random forest and explainable machine learning (SHAP) method to quantitatively estimate the sensitivity of burned area to PPT (see *Random forest and SHAP* for details). Both eliminate the compounding effects of maximum photosynthesis, temperature, total precipitation, radiation, and relative humidity. To mitigate the implication of divergent spatial resolutions among FireMIP models and satellite observations, we calculated the area-weighted mean and 95% confidence interval of sensitivities across the globe.

Random forest and SHAP

We used random forest and explainable machine learning (SHAP) methods to estimate the sensitivity of burned area to PPT for the satellite observations and FireMIP models. In contrast to traditional statistical techniques, the methods integrate the advantages of random forest, encompassing bootstrap aggregation and non-distribution assumption, with the strengths of SHAP, ensuring coherence between global interpretations and local explanations [55, 56]. This method strengthens the robustness of the results from traditional statistics approaches [57].

We conducted this analysis following the ref. [57] through several steps. (i) For each grid cell, we used the year-to-year variations of burned areas as the dependent variables, and the year-to-year variations of PPT, maximum photosynthesis, temperature, total precipitation, radiation, and relative humidity as the predictors, to train the random forest model. The hyperparameter settings for random forest were number of estimators=100; maximum features=30%; random state=42. (ii) SHAP was used to interpret the trained model to calculate the marginal contributions of PPT on burned areas. (iii) The sensitivity of burned area to PPT was estimated as the slope of the relationship between SHAP-derived marginal contributions of PPT and its year-to-year variations by using the Theil-Sen regression.

Uncertainty from anthropogenic fires

Human activity can modify fire dynamics via ignition, active fire suppression, and manipulating the timing and fuel conditions of fires [58, 59]. For example, in the United States, human-caused fire can occur throughout the year with a much longer fire season than natural fires, and occur when climate and fuel conditions are not favorable for burning [60], thereby weakening

the coupling between climate and fire activity [61]. Thus, anthropogenic fires may confound the identification of PPT-fire interactions. However, differentiating between anthropogenic and natural fires remains a challenging task. A recent study compiled a fire-cause reference dataset and applied machine learning technique to predict global patterns of the fractions of fires and burned area related to natural and anthropogenic ignition sources at a large spatial scale. However, due to the limited fire-cause records globally, the fire-cause reference data primarily originated from North America, leading to large uncertainties in other regions. Actually, they did not identify whether a fire is nature- or human-ignited [61].

To partly mitigate the influence of human fire use, we excluded croplands when calculating burned area after PPT within a 0.25° grid cell, as croplands are human-dominated ecosystems (see *MODIS global burned area*). We further examined the impact of anthropogenic fires from three aspects: (i) Human activity often leads to land-use and land-cover changes (LULCC), such as land-cover conversion for agricultural expansion in African and South American tropical savannas and Asian semi-arid grasslands [59], and deforestation in tropical forests [62], etc. Therefore, we used long-term MCD12Q1 land cover type products to identify the 500-m pixels where LULCC occurred during the study period. These pixels were masked when calculating burned area after PPT (Fig. S23). (ii) We applied the global areas of low human impact dataset with 1 km resolution [63] to distinguish human- and nature-dominated regions. We calculated the fraction of low impact area (LIA) within a 0.25° grid cell. Larger LIA indicates low-impact land by human, where fire activity may be mainly determined by natural factors. Lower LIA indicates areas with larger human impact, where fire activity may be largely influenced by human activity (Fig. S24). (iii) We also employed an intact forest landscape data for the year 2000, which can represent the forest and naturally treeless ecosystems with no remotely detected signs of human activity [64]. We compared the effects of PPT on subsequent fire activity among the globe, global intact forest, global areas outside of intact forest, tropical intact forest (25°S-25°N), and extratropical intact forest (Fig. S25).

Supplementary Figures

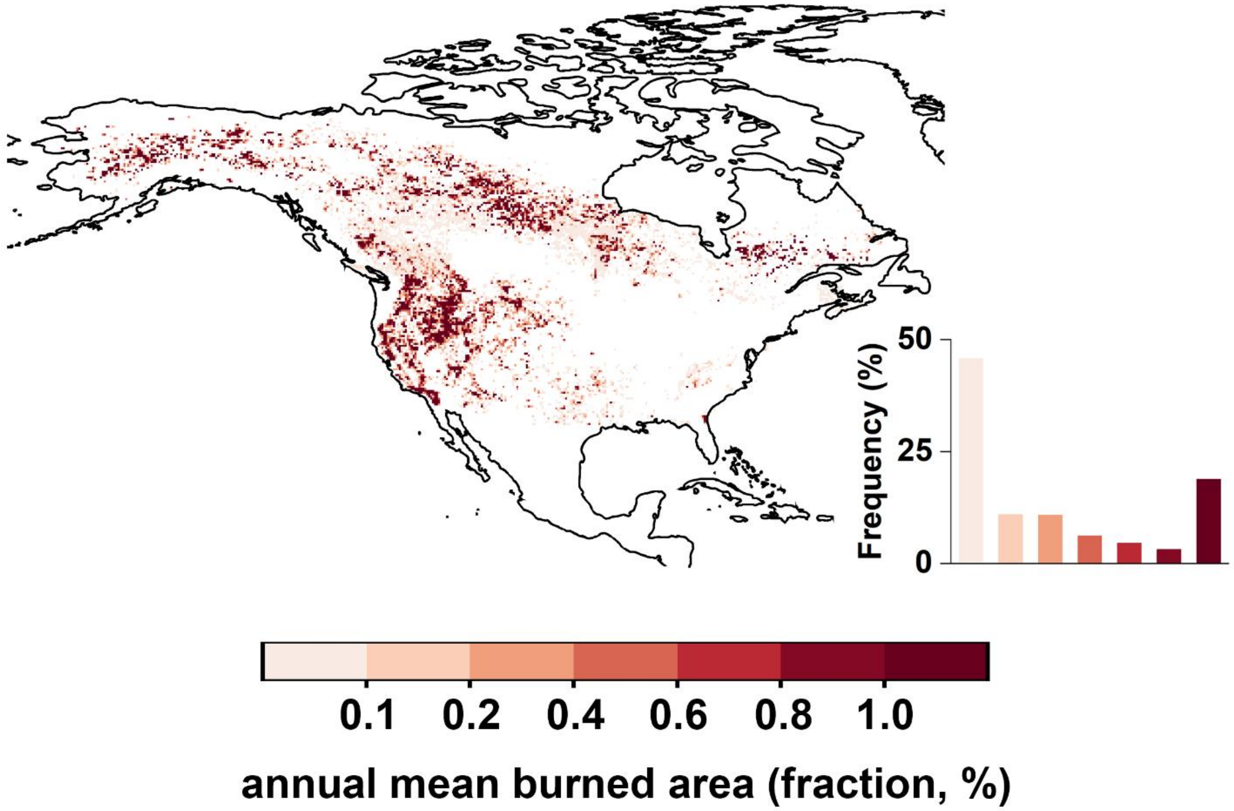

Supplementary Figure 1. Annual mean burned area after CSIF-derived peak photosynthesis timing (PPT) from 2002 to 2018 for fire perimeters data. Fire perimeters data include the National Burned Area Composite (NBAC) for Canada, and Monitoring Trends in Burn Severity (MTBS) for the US. Burned area was expressed as the fraction of a grid cell that burns at 0.25°resolution. (审图号：GS 京 （2024）1632 号）

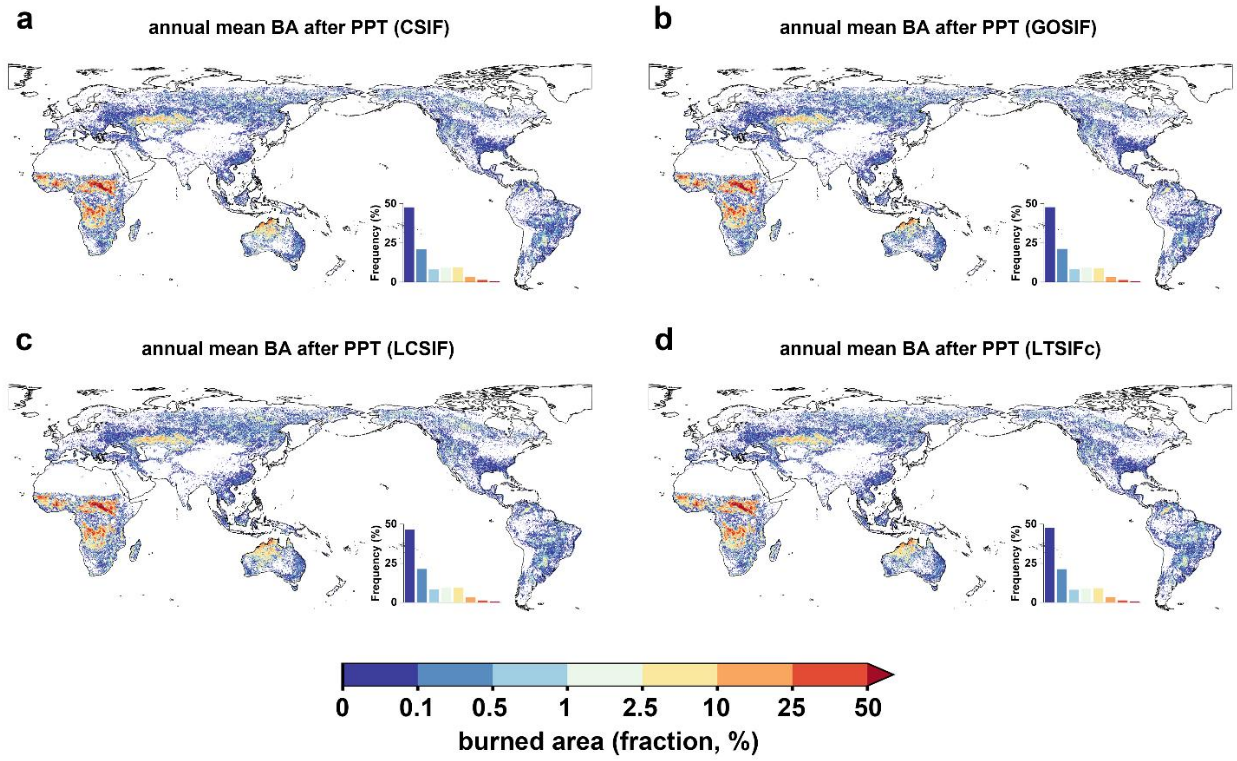

Supplementary Figure 2. Annual mean global burned area after peak photosynthesis timing (PPT) from 2002 to 2018. PPT was derived from CSIF (a), GOSIF (b), LCSIF (c), and LT_SIFc (d). Burned area was derived from MCD64A1 V6. Burned area was expressed as the fraction of a grid cell that burns at 0.25 °resolution. (审图号：GS 京 （2024）1632 号）

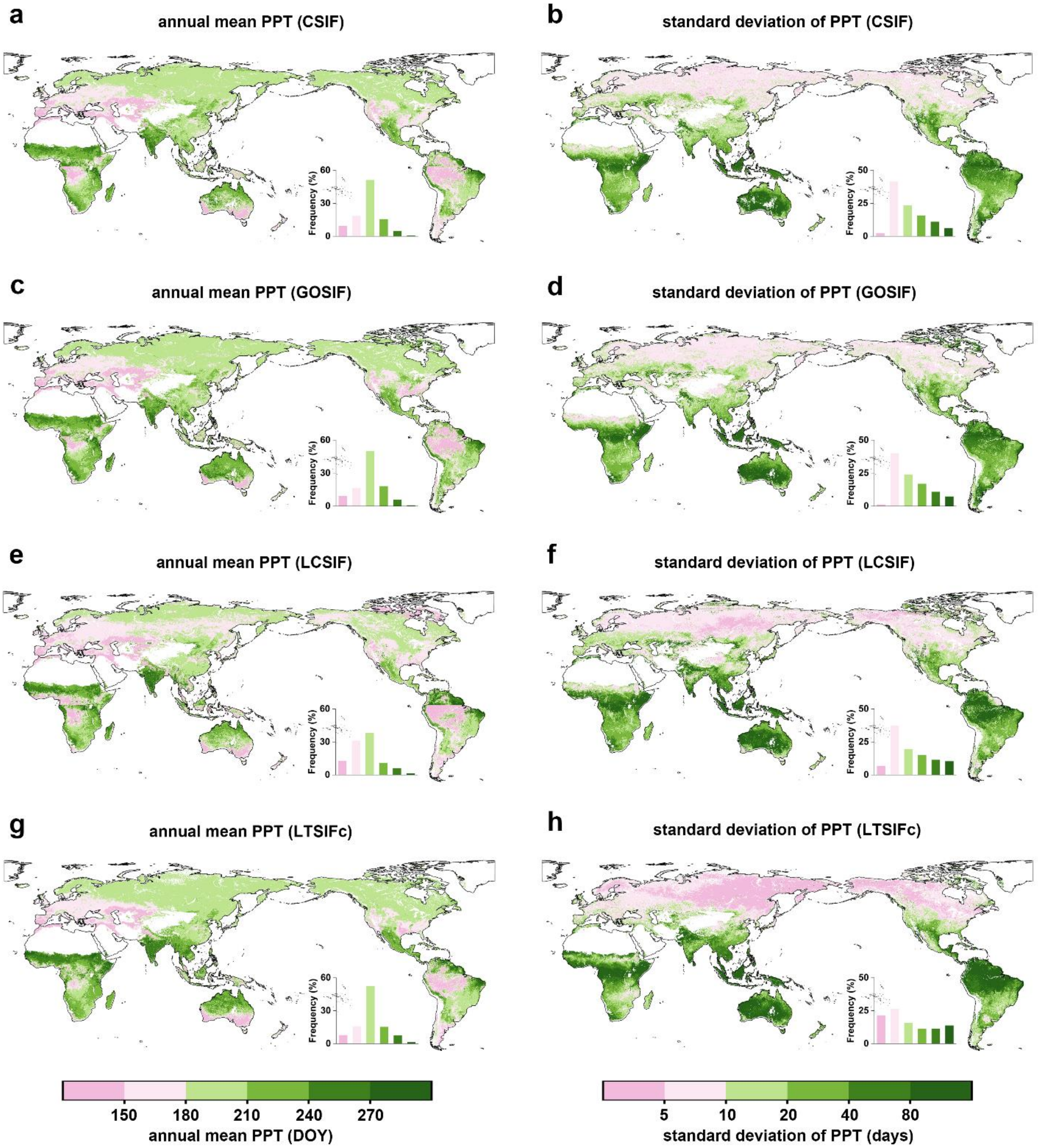

Supplementary Figure 3. Annual mean (left) and standard deviation (right) of global PPT over 2002–2018 for CSIF (a-b), GOSIF (c-d), LCSIF (e-f), and LT_SIFc (g-h). Note that the day of year was identified as from July 1st in the previous year to June 30th in current year for the Southern Hemisphere. (审图号：GS 京 （2024）1632 号）

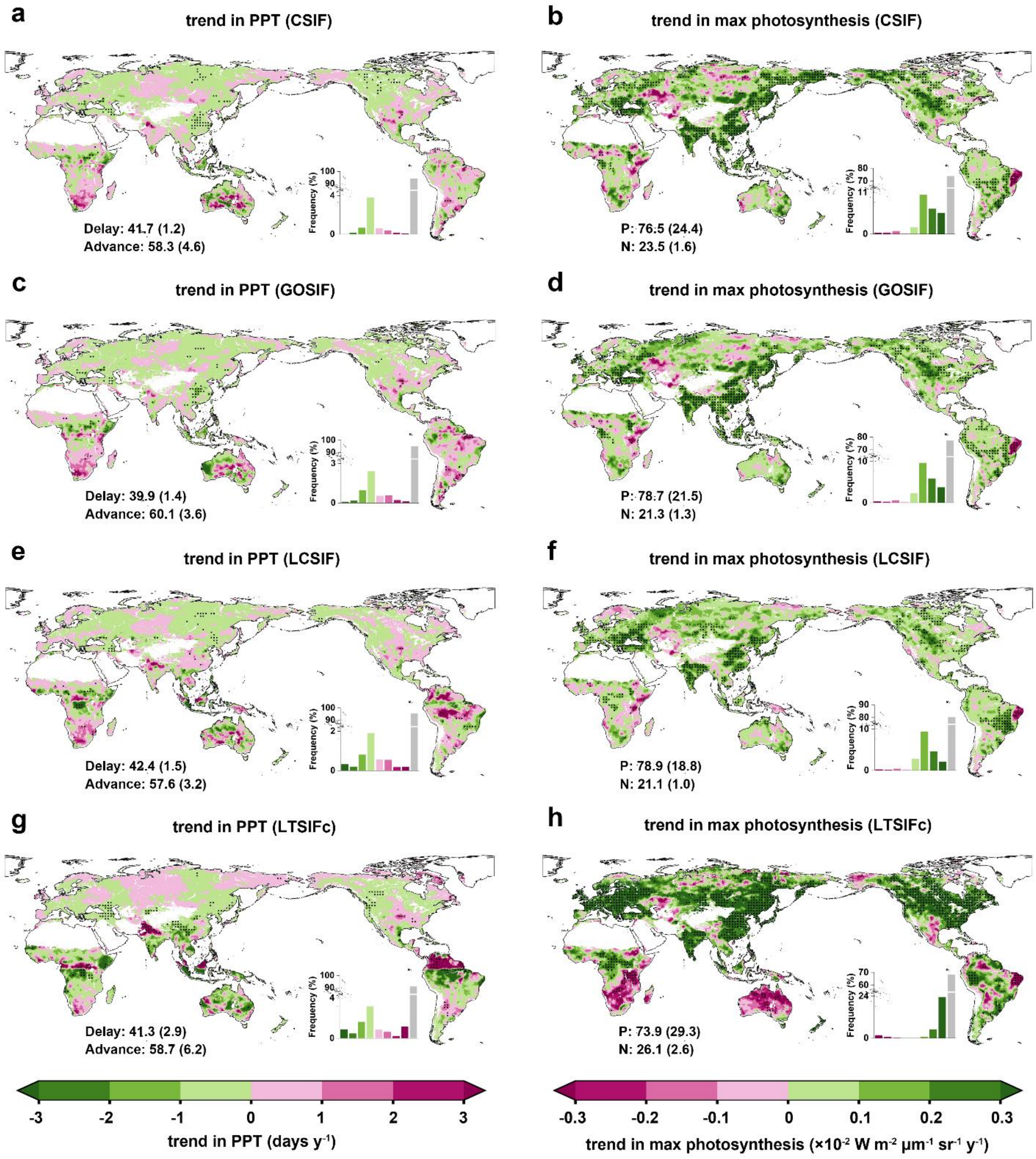

Supplementary Figure 4. Trends in peak photosynthesis timing (PPT; left) and maximum photosynthesis (SIF_{max}; right) over 2002-2018 for CSIF (a-b), GOSIF (c-d), LCSIF (e-f), and LT_SIFc (g-h). Labels in the left indicate the percentage of areas shown delayed and advanced PPT. P and N indicate the percentage of increased and decreased maximum photosynthesis, respectively. Black dots indicate regions with significant trends (p-value < 0.05). (审图号：GS 京 （2024）1632 号)

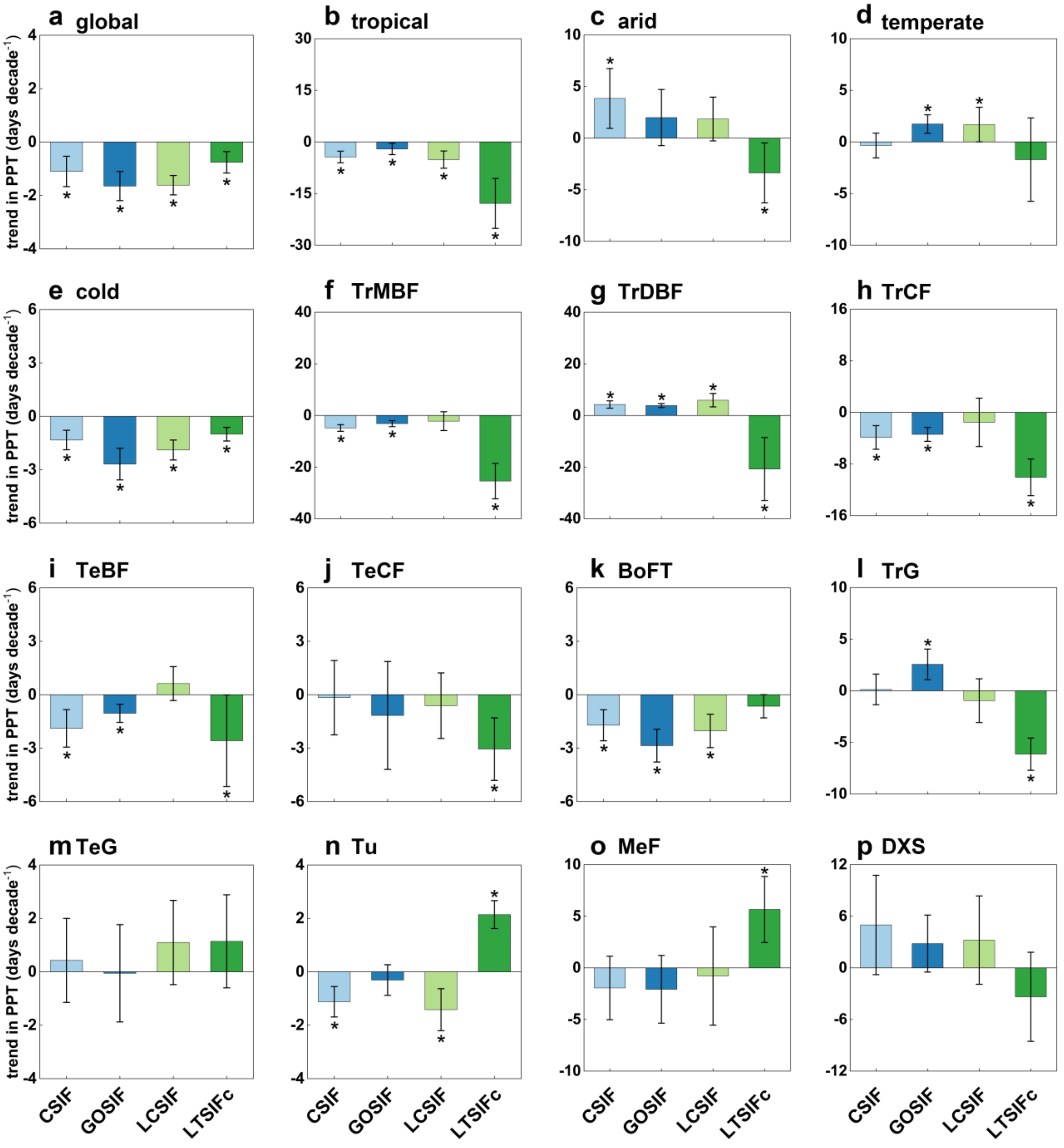

Supplementary Figure 5. Decadal trends in peak photosynthesis timing (PPT) over 2002-2018 derived from CSIF, GOSIF, LCSIF, and LT_SIFc for the globe, 4 climate zones, and 11 biomes. Bar and error bar indicate decadal trend and corresponding 95% confidence interval, respectively, based on linear regression (see *Extraction of global peak photosynthesis timing* for detail). The asterisk denotes p-value < 0.05.

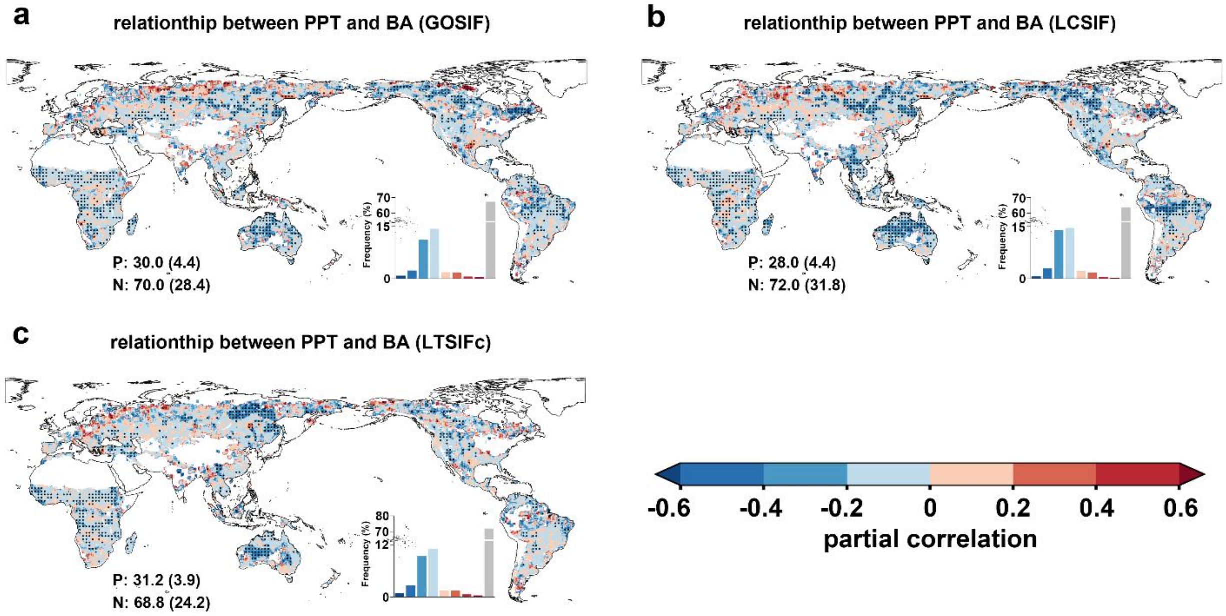

Supplementary Figure 6. Robustness of the relationship between peak photosynthesis timing (PPT) and subsequent burned area. PPT was derived from GOSIF (a), LCSIF (b), and LT_SIFc (c). P and N indicate the percentage of positive and negative correlation, respectively. Black dots indicate regions with significant partial correlations (p-value < 0.05). (审图号：GS 京 （2024）1632 号）

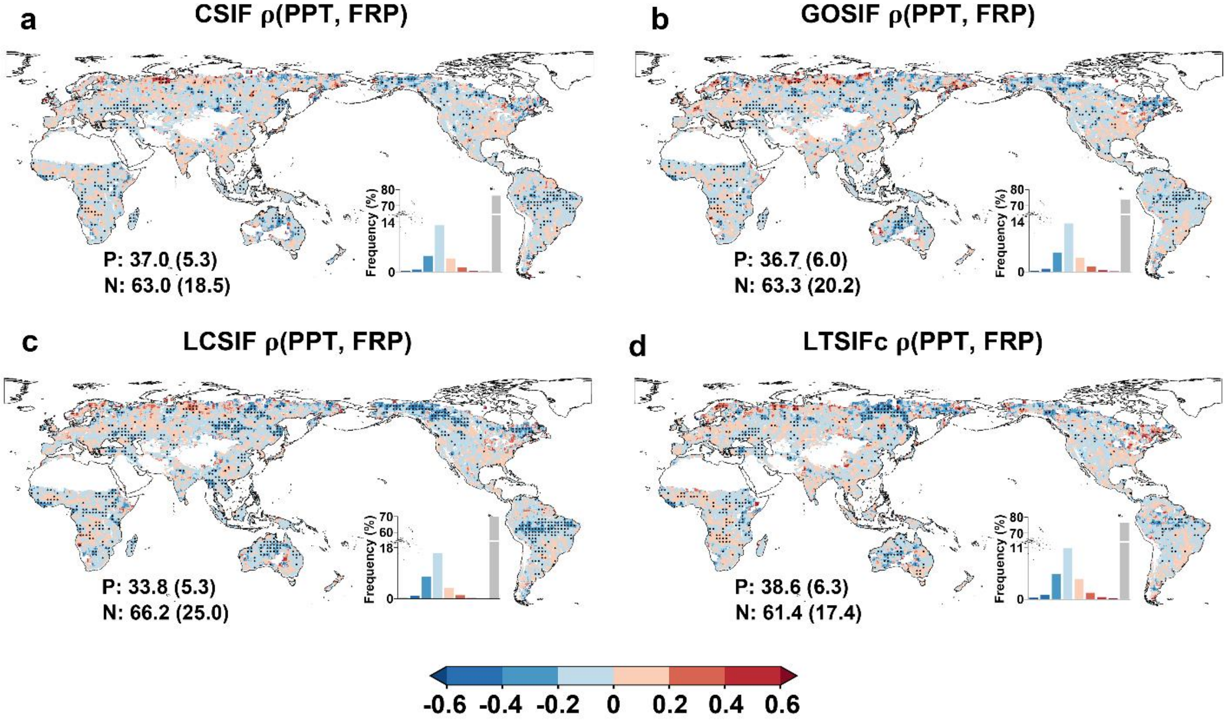

Supplementary Figure 7. The impact of peak photosynthesis timing (PPT) on subsequent fire intensity represented by fire radiative power (FRP) extracted from MCD14ML V6 active fire product. PPT was derived from CSIF (a), GOSIF (b), LCSIF (c), and LT_SIFc (d). P and N indicate the percentage of positive and negative correlation, respectively. Black dots indicate regions with significant partial correlations (*p*-value < 0.05). (审图号：GS 京 （2024）1632 号）

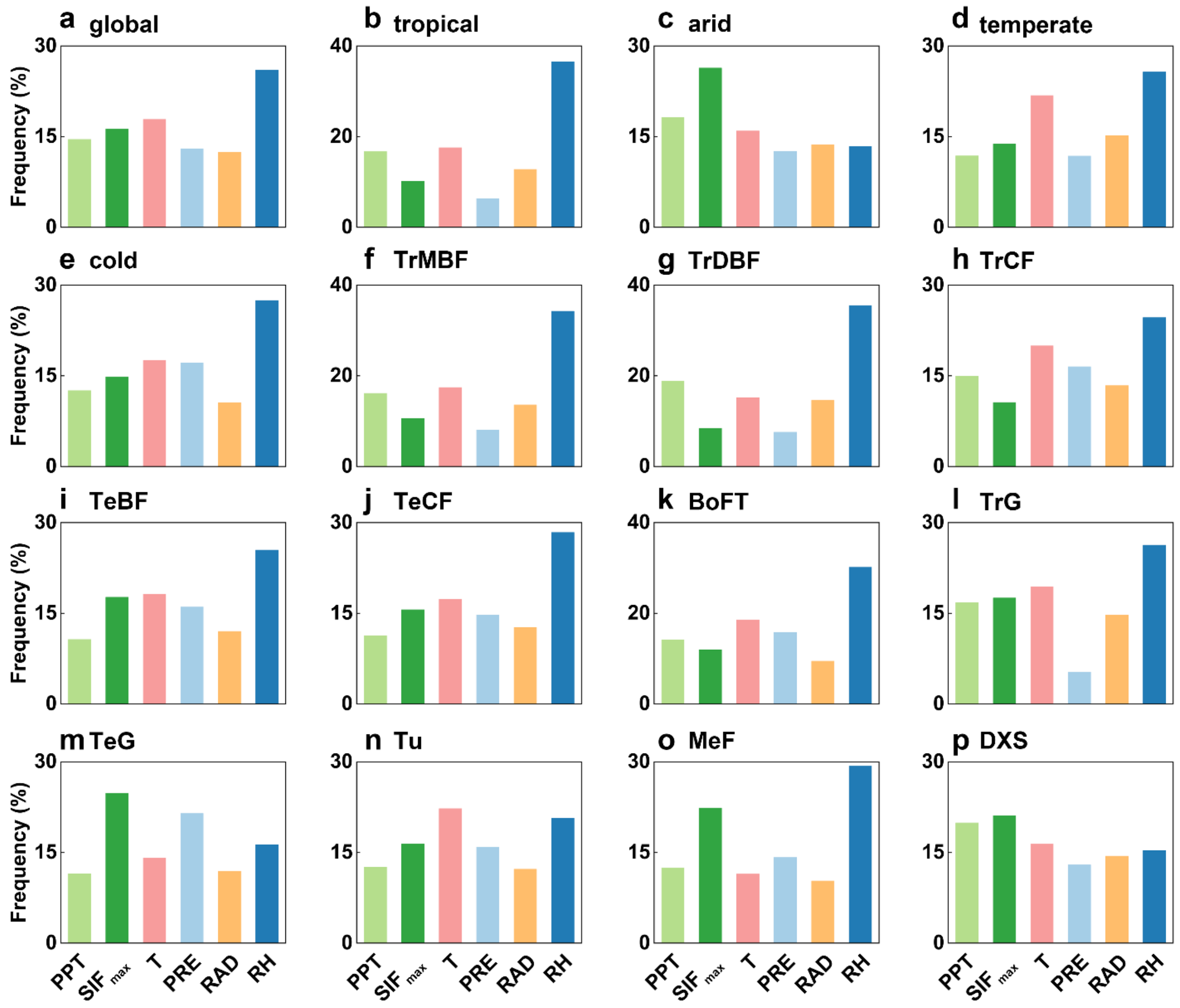

Supplementary Figure 8. Regional frequency of the most important factor in controlling variation of post-PPT burned area based on CSIF-derived PPT and MODIS burned area data for the globe, 4 climate zones, and 11 biomes. The most important factor for each grid cell was determined by the absolute value of partial correlation between each factor and post-PPT burned area. When calculating the partial correlation between each factor and burned area, we excluded the effects of the remaining factors. PPT: peak photosynthesis timing; SIF_{max}: maximum photosynthesis; T: surface air temperature; PRE: total precipitation; RAD: downwards surface solar radiation; RH: relative humidity.

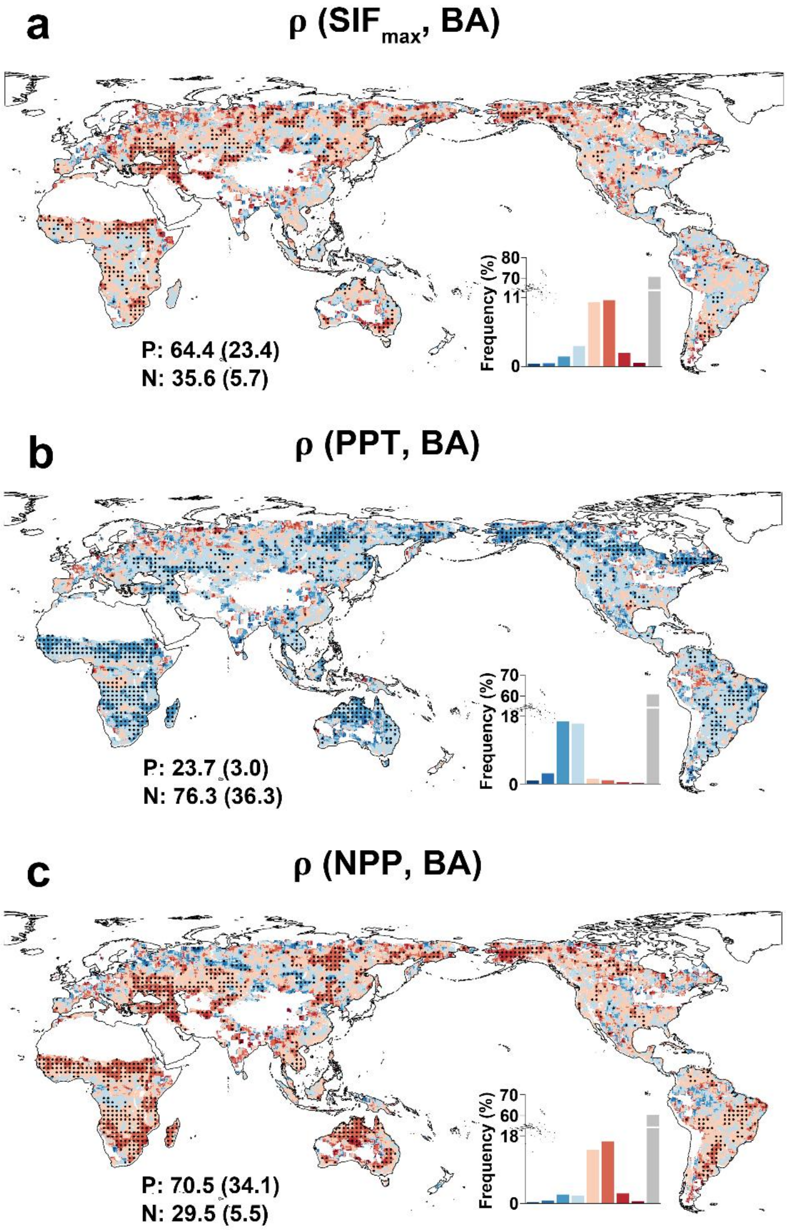

Supplementary Figure 9. (a) Partial correlation between CSIF-derived SIF_{max} and burned area. Same as Fig. 2d, partial correlation was conducted based on MODIS-derived burned area, CSIF-derived PPT and SIF_{max}, temperature (T), total precipitation (PRE), solar radiation (RAD), and relative humidity (RH). (b) Partial correlation between CSIF-derived PPT and burned area. (c) Same as (b) but for the pre-PPT accumulated NPP. When conducting partial correlations in (b-c), we replaced SIF_{max} with NPP. P and N indicate the percentage of positive and negative correlation, respectively. Black dots indicate regions with significant partial correlations (p-value < 0.05). (审图号：GS 京 （2024）1632 号）

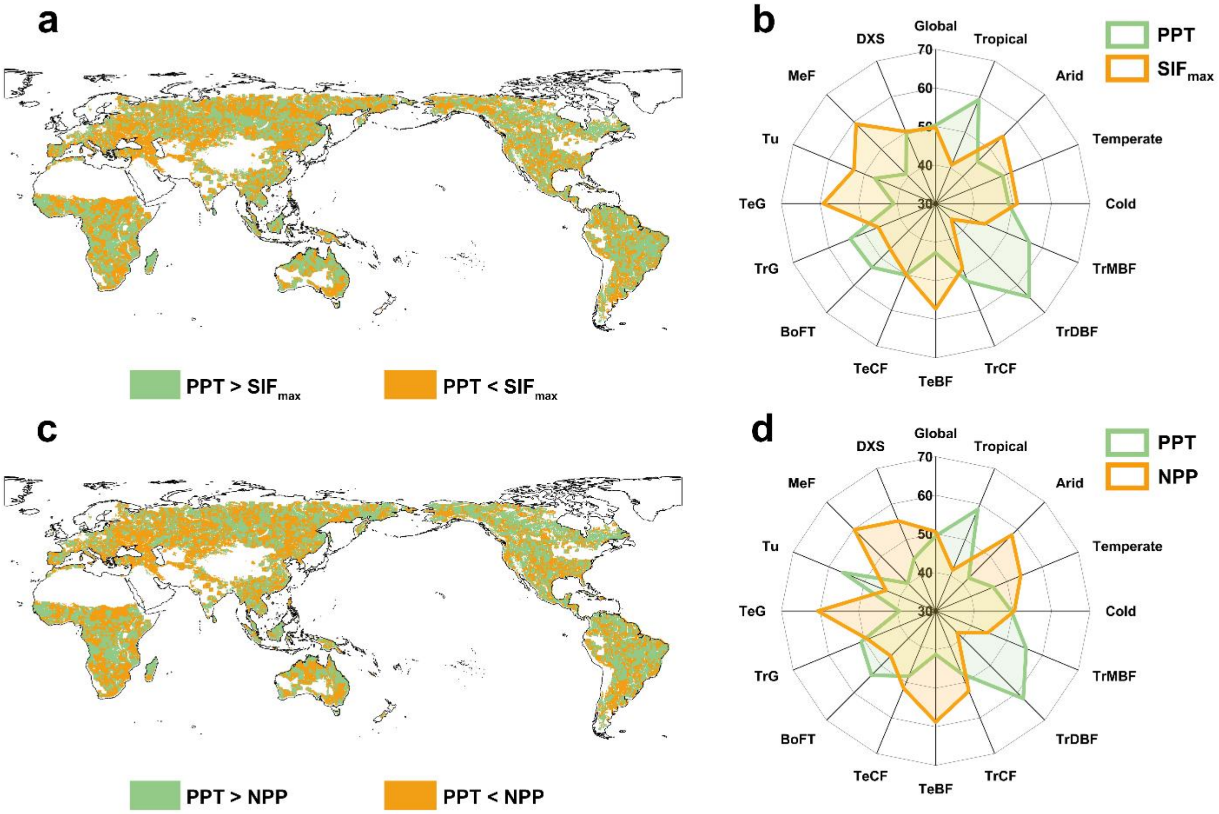

Supplementary Figure 10. Relative importance of PPT and SIF_{max} (a-b) or pre-PPT accumulated NPP (c-d) in controlling post-PPT burned area. The relative importance is determined based on the absolute value of partial correlation. PPT and SIF_{max} are extracted from CSIF product. (b) and (d) show the dominant area fraction of PPT and SIF_{max} (or NPP) for the globe, 4 climate zones, and 11 biomes. (审图号：GS 京 （2024）1632 号）

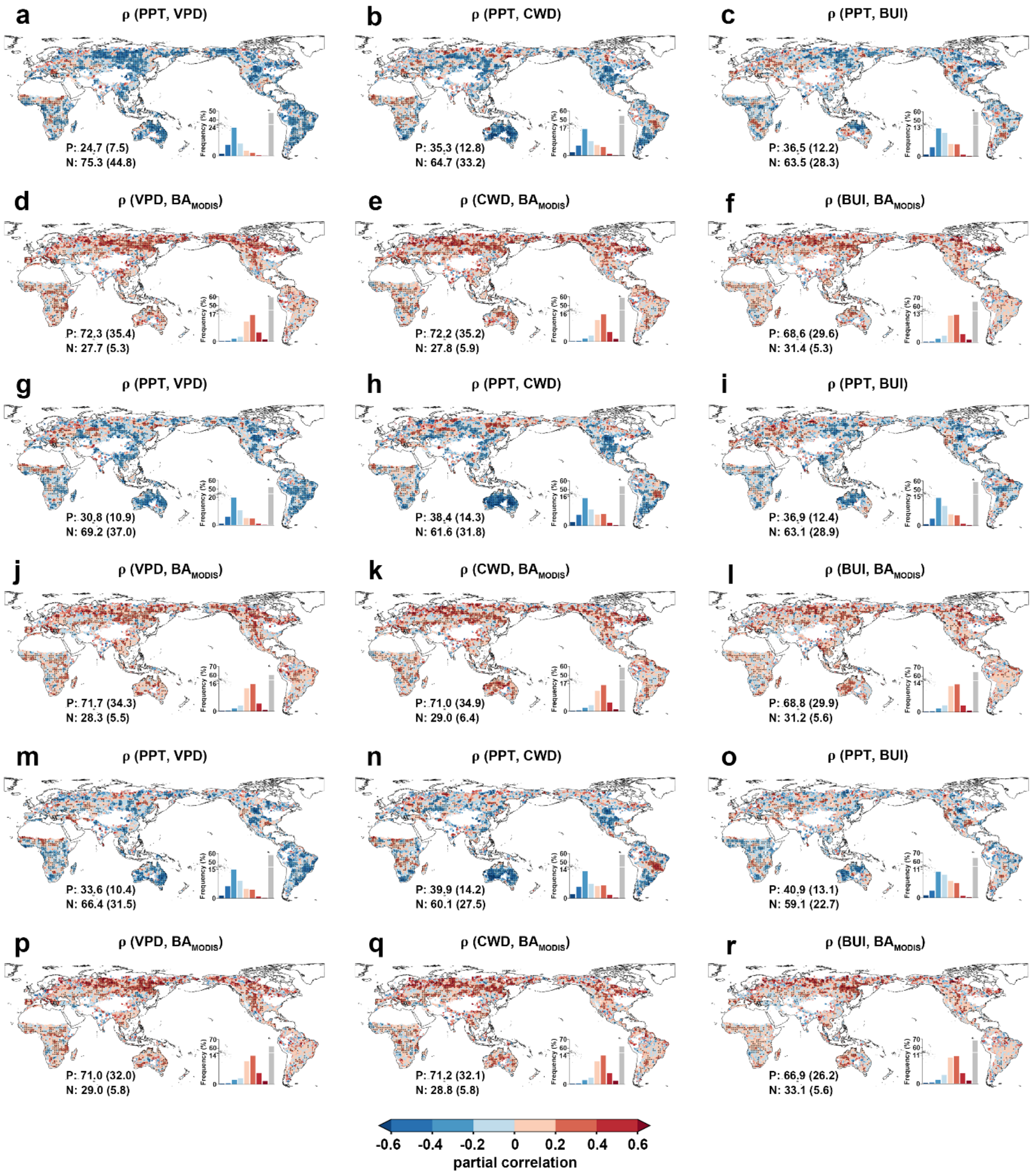

Supplementary Figure 11. Robustness of the underlying mechanisms linking peak photosynthesis timing (PPT) and burned area. PPT was derived from GOSIF (a-f), LCSIF (g-l), and LT_SIFc (m-r). Global patterns of partial correlations between PPT and factors (VPD, CWD, and BUI during the potential fire season after PPT), and then between factors and burned area. Black dots indicate regions with significant partial correlations (p-value < 0.05). P and N indicate the percentage of positive and negative correlations, respectively. (审图号：GS 京（2024）1632号）

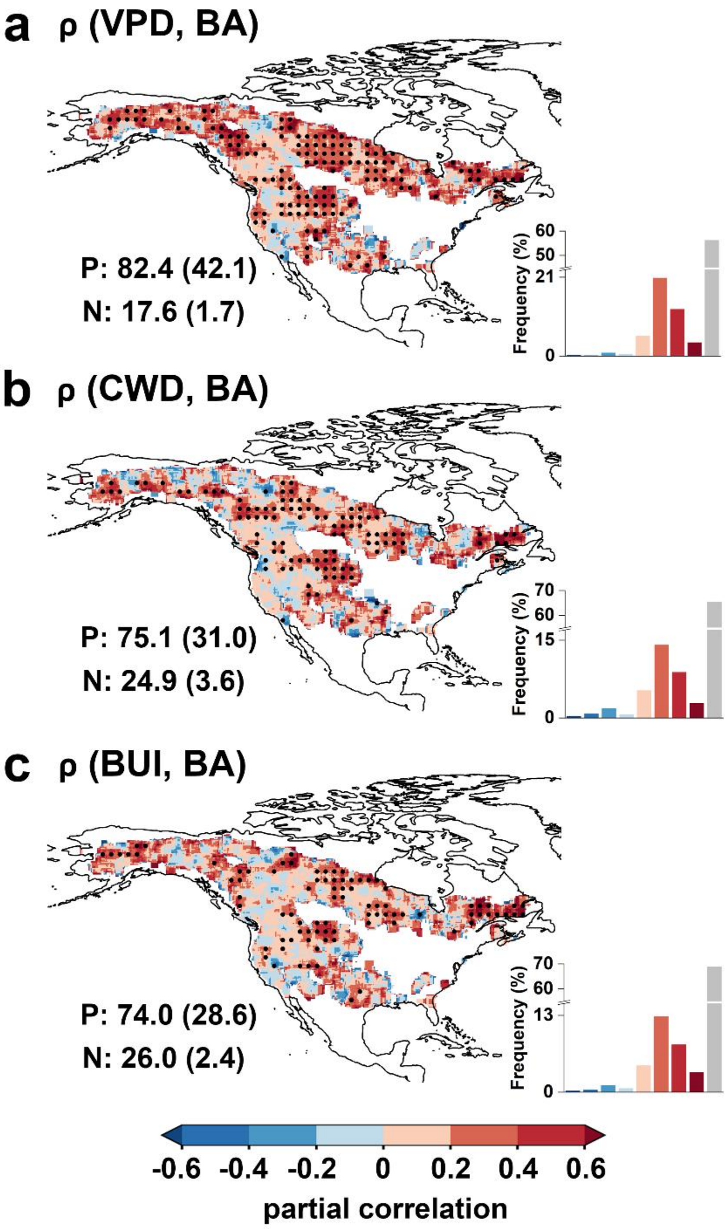

Supplementary Figure 12. Relationships between explainable factors and burned area (BA) for fire perimeters data. Partial correlations of VPD versus BA (a), CWD versus BA (b), and BUI versus BA (c). The explainable factors indicate the climate conditions during the potential fire season after CSIF-derived PPT. Black dots indicate regions with significant partial correlations (*p*-value < 0.05). P and N indicate the percentage of positive and negative correlations, respectively. (审图号：GS 京 （2024）1632 号）

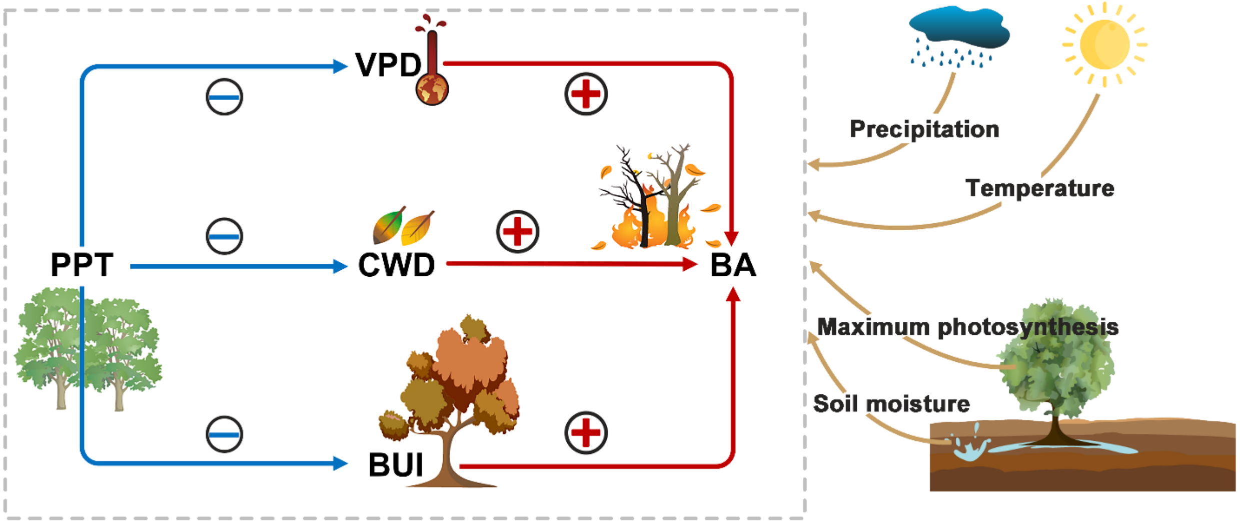

Supplementary Figure 13. Schematic path diagram showing the effect of peak photosynthesis timing (PPT) on the subsequent burned area through changing atmospheric aridity (VPD), plant water stress (CWD), and potential fuel availability (BUI). This path diagram contains three hypothetical pathways: PPT-VPD-BA, PPT-CWD-BA, and PPT-BUI-BA. The model also considers the effect of some post-PPT climatic (temperature, precipitation, and soil moisture) and biological (maximum photosynthesis) factors on VPD, CWD, BUI, and burned area. The + and – in circle indicate positive (red) and negative (blue) bivariate correlation, respectively. PPT: peak photosynthesis timing; VPD: vapor pressure deficit; CWD: climatic water deficit; BUI: build up index; BA: burned area after PPT. These climatic variables were characterized over the period from the month of PPT to November, representing the conditions of potential fire season after PPT (see *Characterization of climate conditions of potential fire season after PPT* for detail).

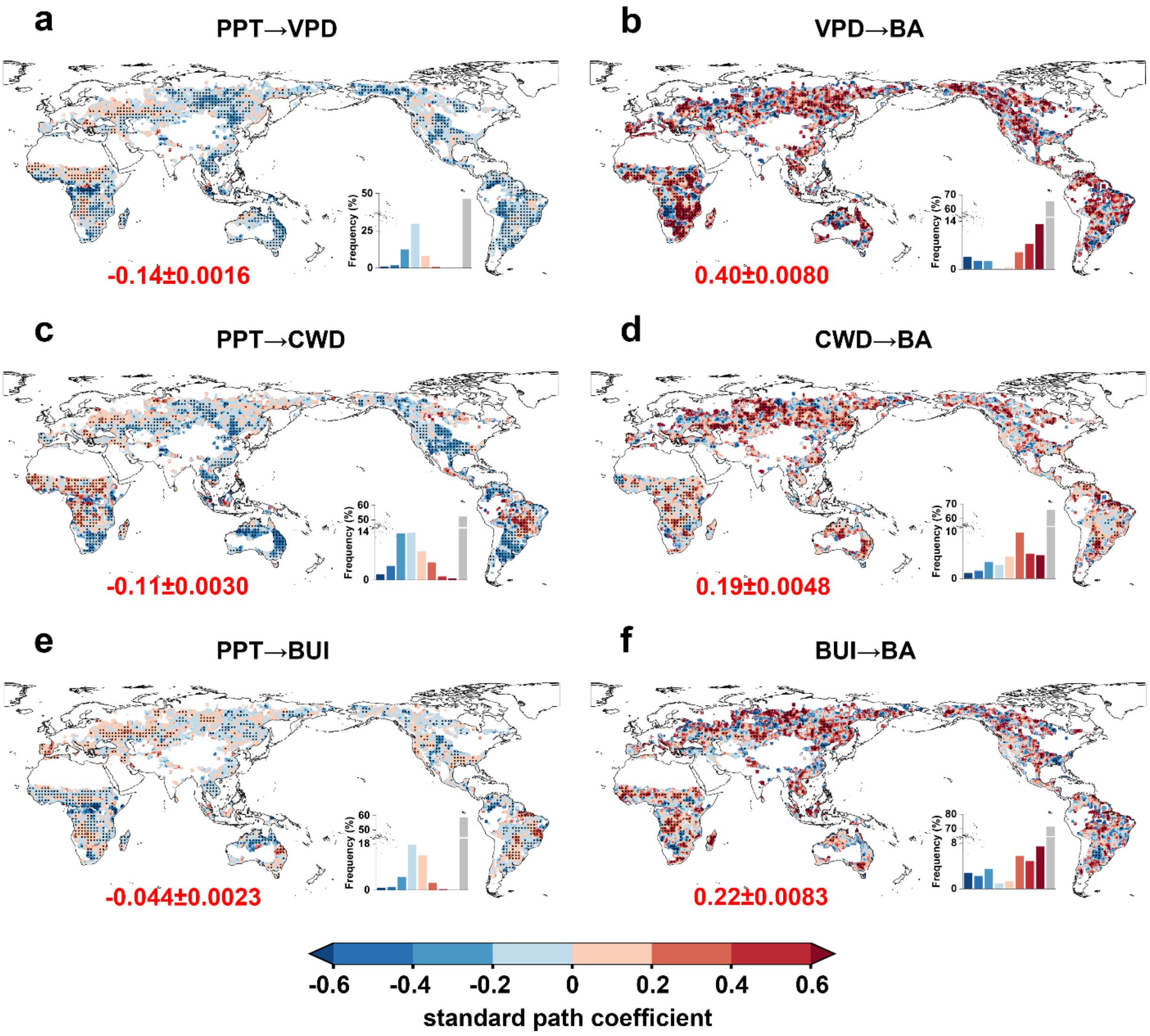

Supplementary Figure 14. Standard path coefficient of each path for CSIF-derived peak photosynthesis timing (PPT) and MODIS-derived burned area data. “X-Y” indicates the effect of X on Y. Black dot indicates the standard path coefficient is significant (*p*-value < 0.05). Red label indicates the regional mean ±95% confidence interval of standard path coefficient considering the goodness of fit of the model (Fig. S28) and the significance of path coefficient. PPT: peak photosynthesis timing; VPD: vapor pressure deficit; CWD: climatic water deficit; BUI: build up index; BA: burned area after PPT. (审图号：GS 京 （2024）1632 号）

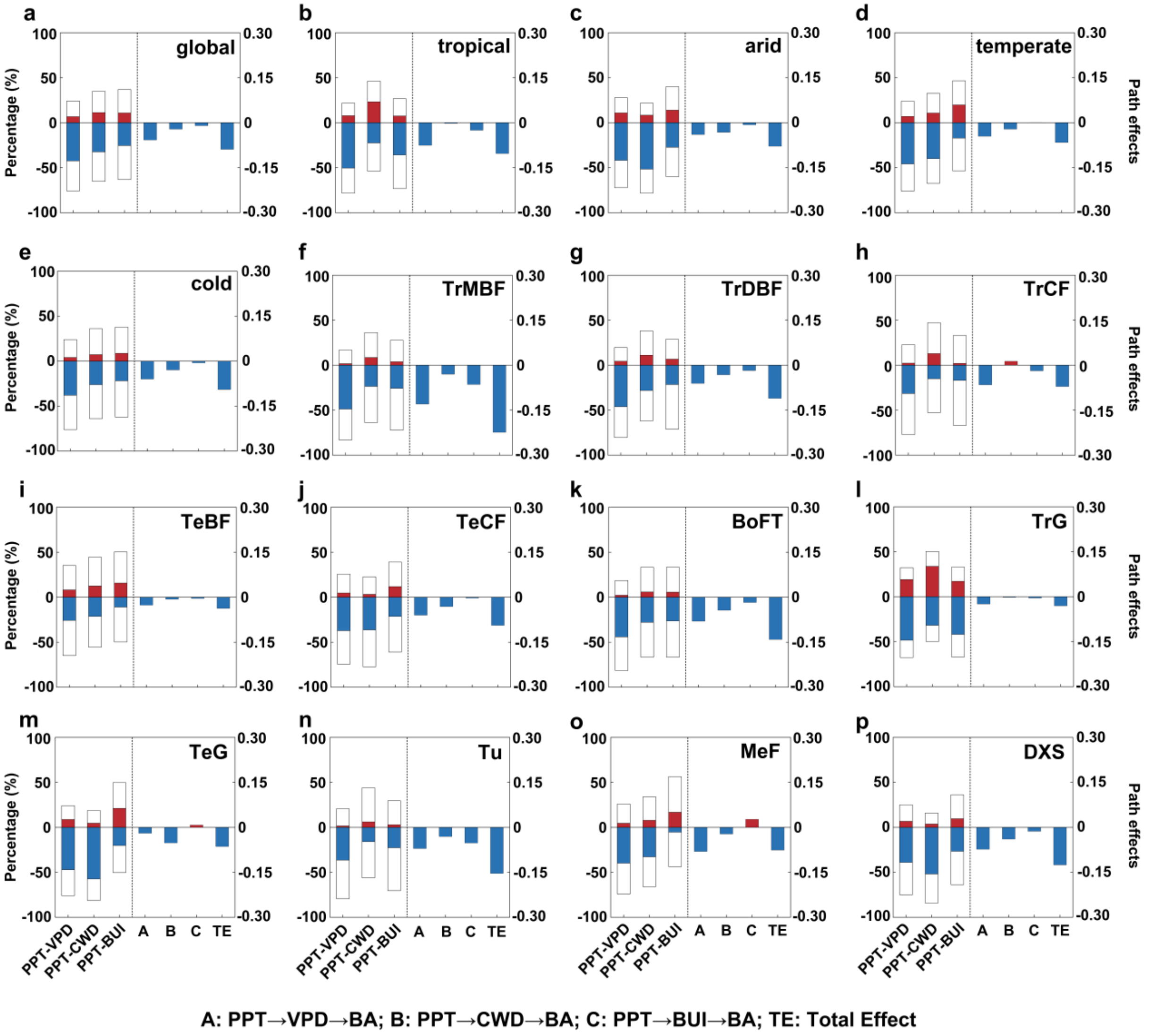

Supplementary Figure 15. The underlying mechanisms linking CSIF-derived peak photosynthesis timing (PPT) and subsequent MODIS-derived burned area for the globe, 4 climate zones, and 11 biomes. The boxes show the percentage of significant (p-value < 0.05) positive (red) and negative (blue) partial correlations for PPT-VPD, PPT-CWD, and PPT-BUI (left; white indicates the percentage of non-significance), and the path effects for Path A, B, C, and total effect based on the structural equation model (right; red and blue bars indicate positive and negative path effects, respectively).

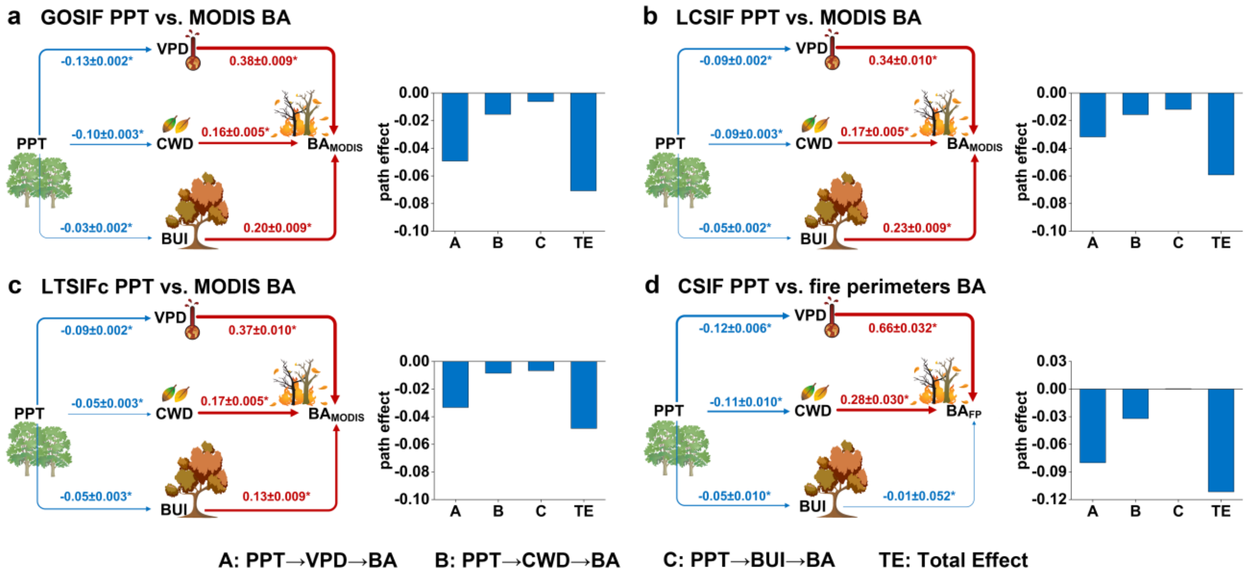

Supplementary Figure 16. Robustness of the underlying mechanisms linking peak photosynthesis timing (PPT) and subsequent burned area using structural equation model (SEM) for GOSIF (a), LCSIF (b), LT_SIFc (c), and fire perimeters (d). Path diagrams and path effects for the mechanisms are shown. The numbers in the path diagram represent the global means and 95% confidence intervals of standardized path coefficients, the asterisks indicate the path coefficients are significant (p-value < 0.05) and the colors (red and blue arrows represent positive and negative effects, respectively) and widths of the arrows represent the signs and magnitudes of the path coefficients, respectively. The color of bar represents positive (red) and negative (blue) path effect, respectively.

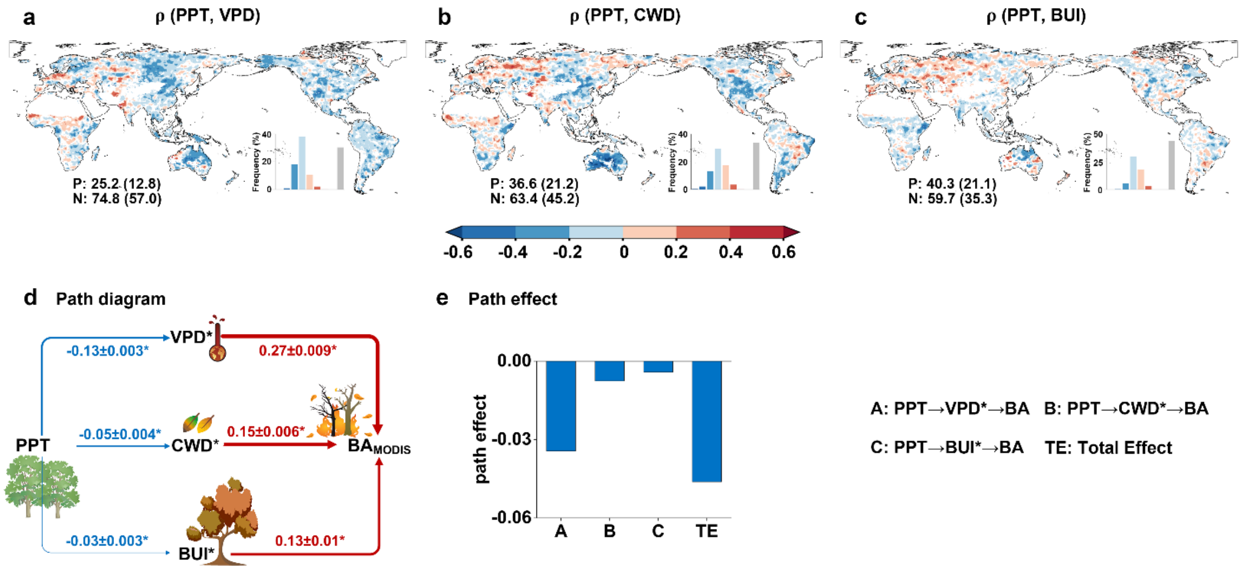

Supplementary Figure 17. Causality networks for the potential mechanisms underlying the linkage between CSIF-derived peak photosynthesis timing (PPT) and MODIS-derived burned area (BA) that further eliminate the effects of pre-PPT climate conditions. The temporal autocorrelations between the climate conditions before and after PPT may confound the identification of the climate feedback arising from advanced PPT. Therefore, we conducted the partial correlations that exclude the effects of the climate variations (temperature and total precipitation) before PPT, maximum photosynthesis, and the climate conditions (temperature, total precipitation, and surface soil moisture) after PPT (a-c). Moreover, we used the VPD*, CWD*, and BUI* that have excluded the effect of pre-PPT temperature and total precipitation from original factors by using random forest, to construct the structural equation model. (d and e) Path diagram and path effect of the SEM. The numbers in the path diagram represent the global means and 95% confidence intervals of standardized path coefficients, the asterisks indicate the path coefficients are significant (p-value < 0.05) and the colors (red and blue arrows represent positive and negative effects, respectively) and the widths of the arrows represent the signs and magnitudes of the path coefficients, respectively. (审图号：GS 京 （2024）1632 号）

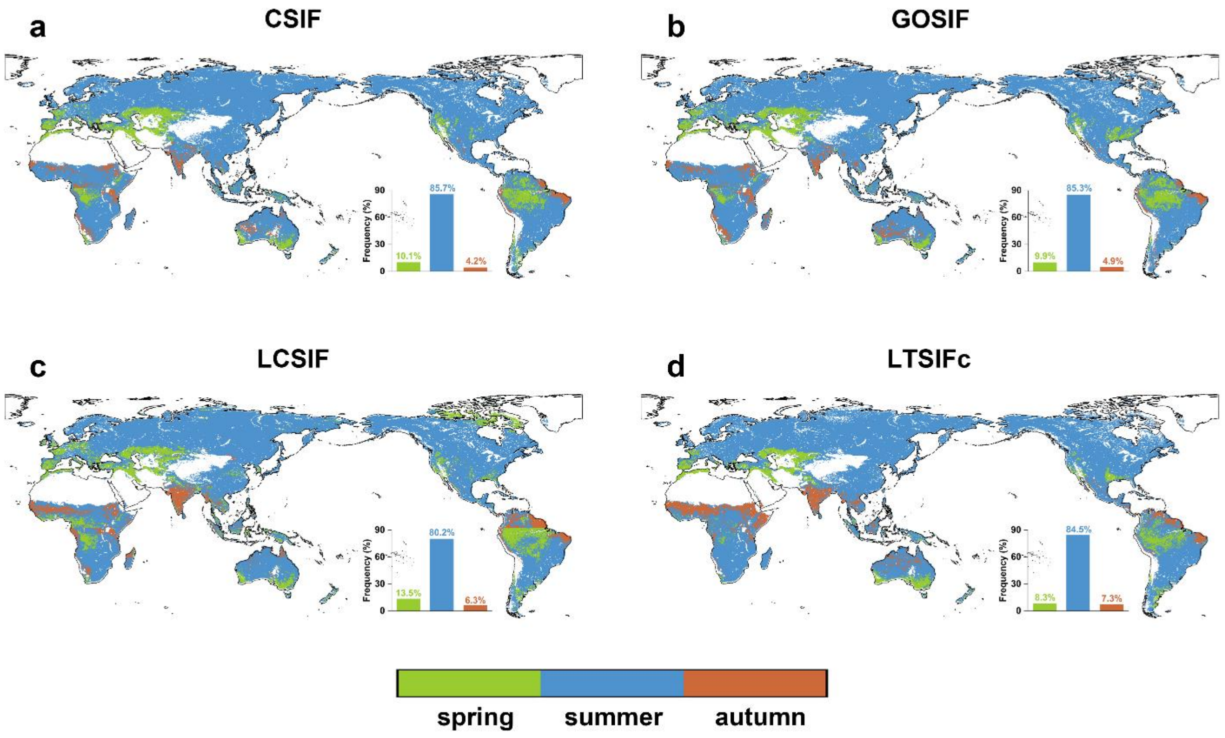

Supplementary Figure 18. The seasons of peak photosynthesis timing for CSIF (a), GOSIF (b), LCSIF (c), and LT_SIFc (d). The colored labels indicate the percentages of months in spring (green), summer (blue), and autumn (orange). It shows that for the majority of areas, PPT occurs in summer. (审图号：GS 京 （2024）1632 号）

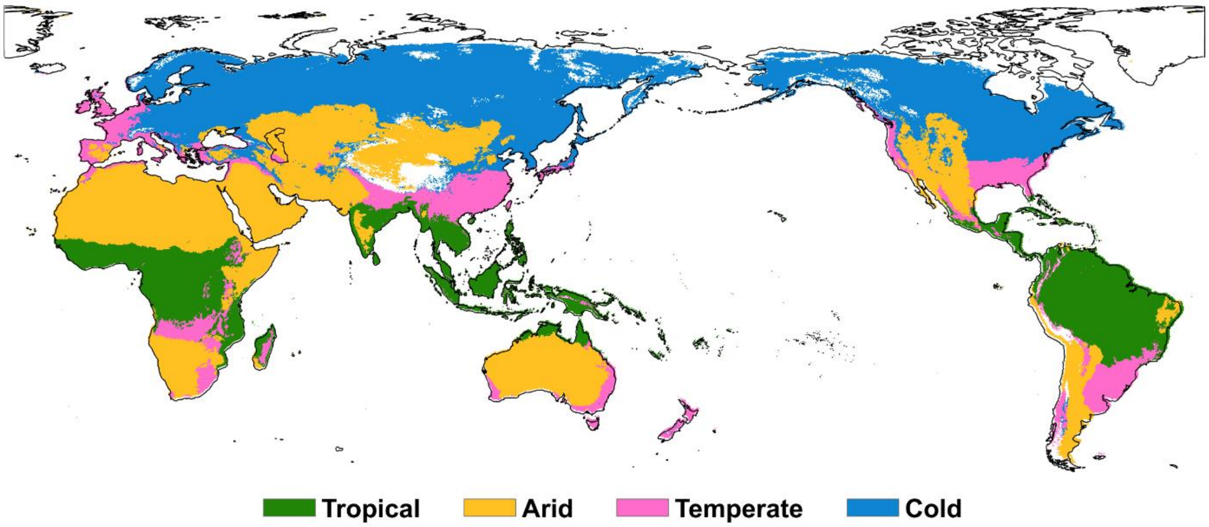

Supplementary Figure 19. Global map of tropical, arid, temperate, and cold regions derived from Köppen-Geiger climate classification map. Polar was excluded in our study. (审图号：GS 京（2024）1632 号）

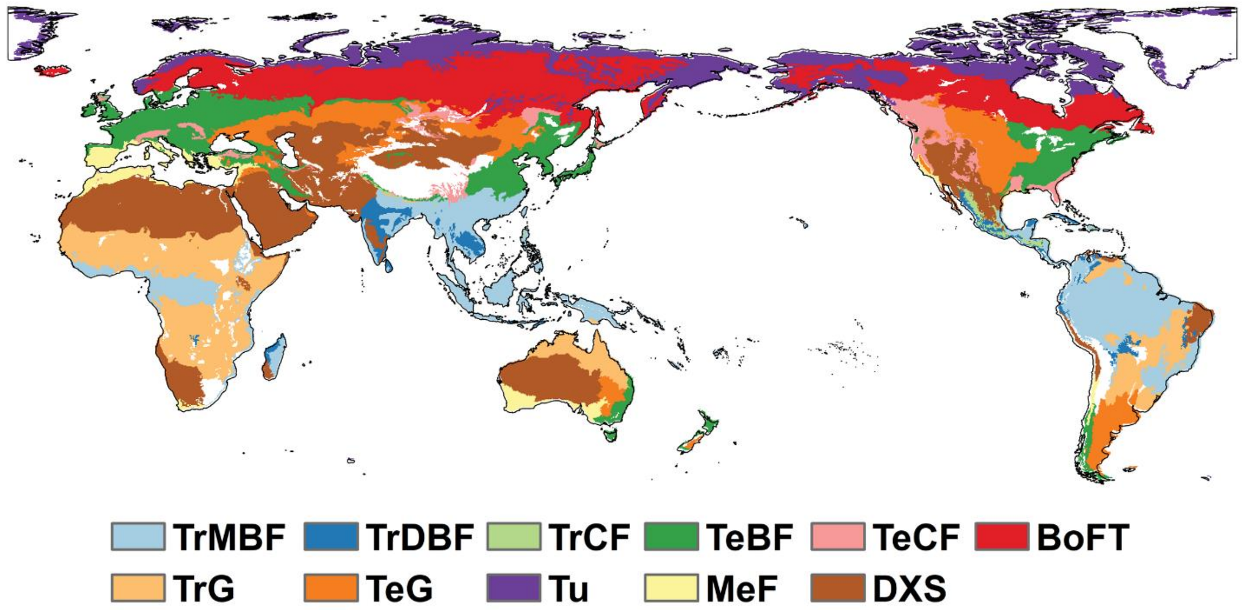

Supplementary Figure 20. Global map of biomes derived from the Terrestrial Ecoregions of the World. TrMBF: Tropical and Subtropical Moist Broadleaf Forests; TrDBF: Tropical and Subtropical Dry Broadleaf Forests; TrCF: Tropical and Subtropical Coniferous Forests; TeBF: Temperate Broadleaf and Mixed Forests; TeCF: Temperate Coniferous Forests; BoFT: Boreal Forests/Taiga; TrG: Tropical and Subtropical Grasslands, Savannas and Shrublands; TeG: Temperate Grasslands, Savannas and Shrublands; Tu: Tundra; MeF: Mediterranean Forests, Woodlands and Scrub; DXS: Deserts and Xeric Shrublands. (审图号：GS 京 （2024）1632 号）

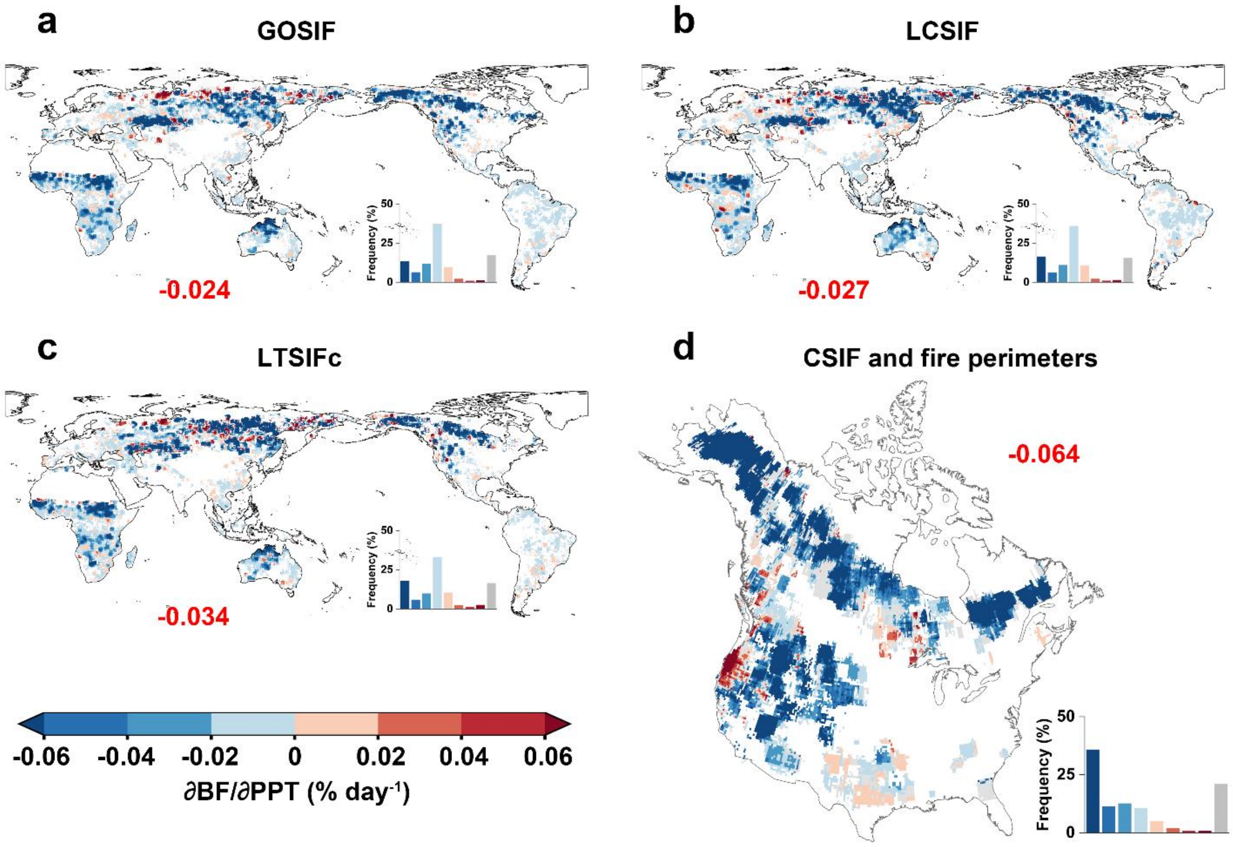

Supplementary Figure 21. Robustness of the sensitivity of burned area to peak photosynthesis timing (PPT) for GOSIF (a), LCSIF (b), LTSIFc (c), and fire perimeters (d) based on random forest and explainable machine learning (SHAP) methods. Gray area indicates the sensitivity is not statistically significant (*p*-value > 0.05). Red label indicates the area-weighted mean of global sensitivity. (审图号：GS 京 （2024）1632 号）

a

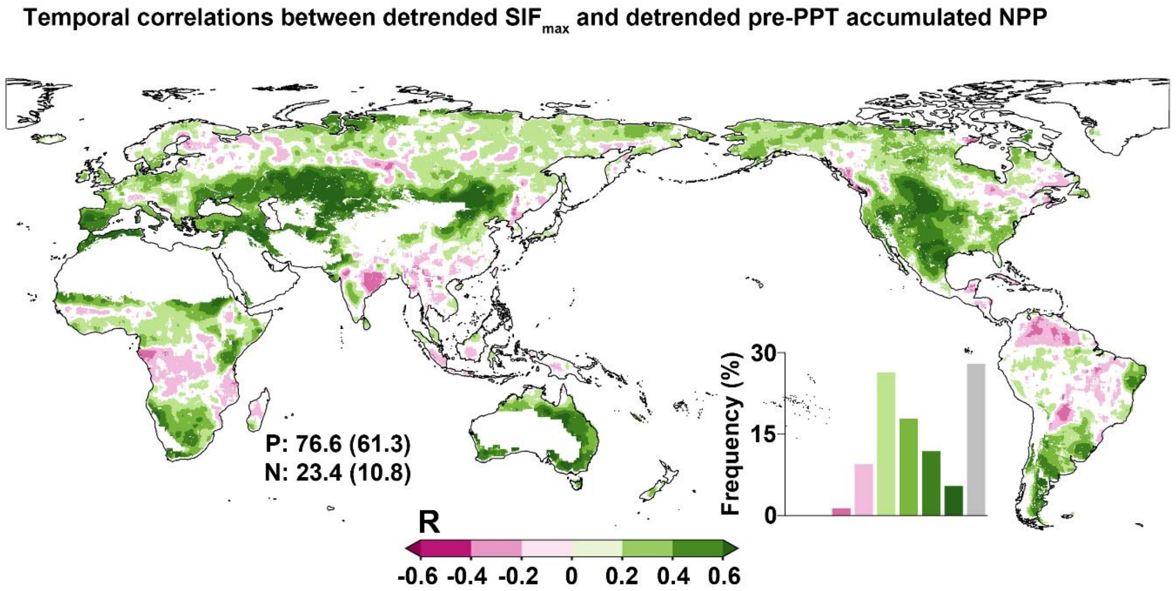

b

Spatial correlations between annul mean SIF_{max} and pre-PPT accumulated NPP, 2002-2018											
Biome	TrMBF	TrDBF	TrCF	TeBF	TeCF	BoFT	TrG	TeG	Tu	MeF	DXS
N	24795	3747	951	20476	6339	35977	24415	17258	19036	3983	15464
R	0.28***	0.53***	0.62***	0.39***	0.41***	0.41***	0.67***	0.88***	0.71***	0.76***	0.84***

Supplementary Figure 22. The spatiotemporal correlations between CSIF-derived maximum photosynthesis (SIF_{max}) and pre-PPT accumulated net primary productivity (NPP). (a) Temporal Spearman correlation (R) between detrended SIF_{max} and detrended NPP at grid cell scale over the period 2002-2018. The white area indicates non-vegetated area or area with non-significant correlation. (b) Spatial correlations (R) between annual mean SIF_{max} and NPP for 11 biomes (Fig. S20). N indicates the sample size. *** indicates p-value < 0.01. (审图号：GS 京 （2024）1632 号）

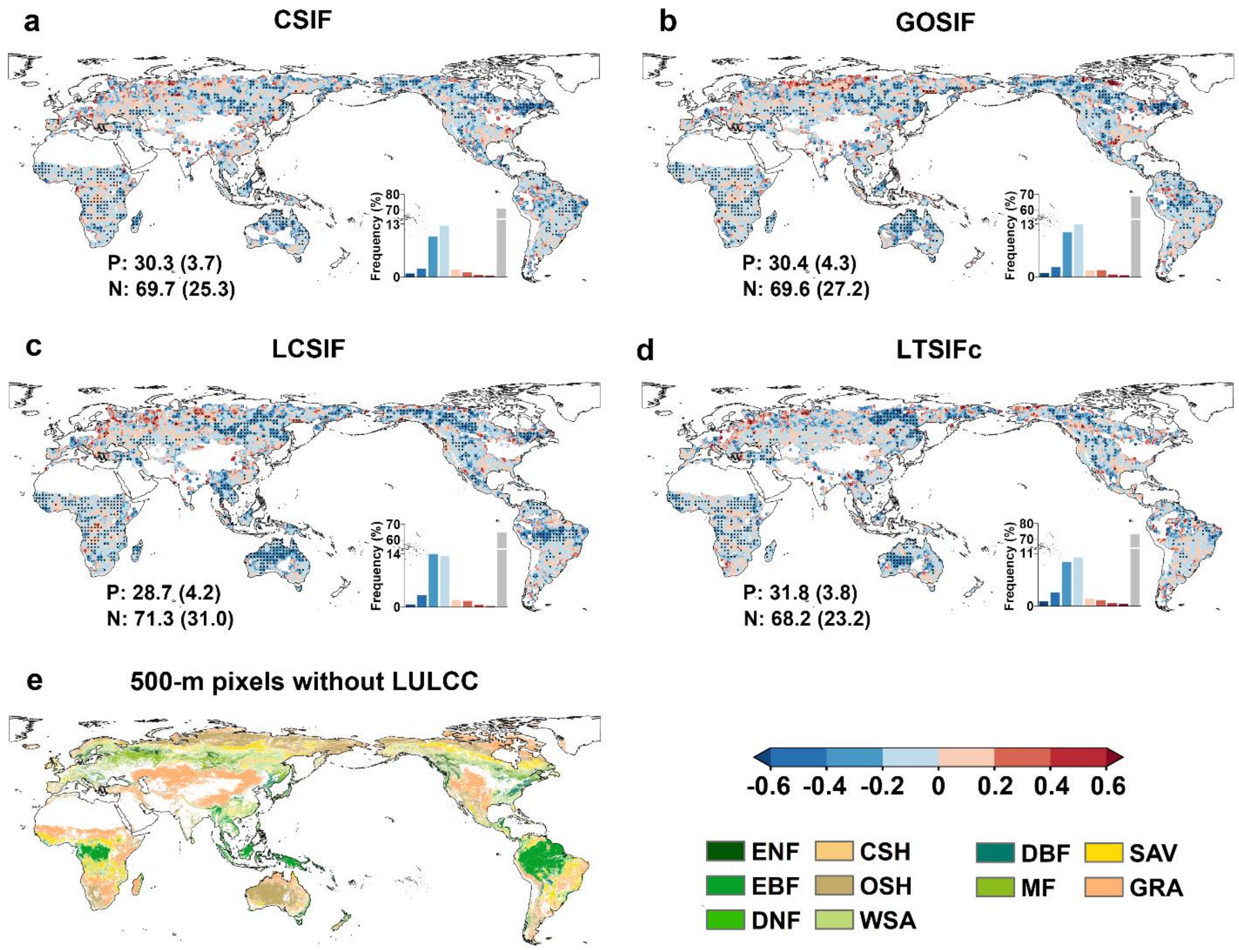

Supplementary Figure 23. Robustness of the relationship between peak photosynthesis timing (PPT) and subsequent burned area after mitigating the effects of land-use and land-cover changes (LULCC). PPT was derived from CSIF (a), GOSIF (b), LCSIF (c), and LT_SIFc (d). P and N indicate the percentage of positive and negative correlation, respectively. Black dots indicate regions with significant partial correlations (*p*-value < 0.05). (e) Global map of areas without LULCC during 2001-2018 based on the 500-m MCD12Q1 V6 land cover type products. Areas with LULCC during the study period, non-vegetated areas, and croplands were removed and shown as white. Human activities often lead to LULCC, e.g. tropical deforestation, agricultural expansion, and livestock grazing, which could directly affect fire activity through fire ignition or suppression. Therefore, removing areas with LULCC can to some extend mitigate the impact of anthropogenic fires on our results (see Methods). (审图号：GS 京 （2024）1632 号）

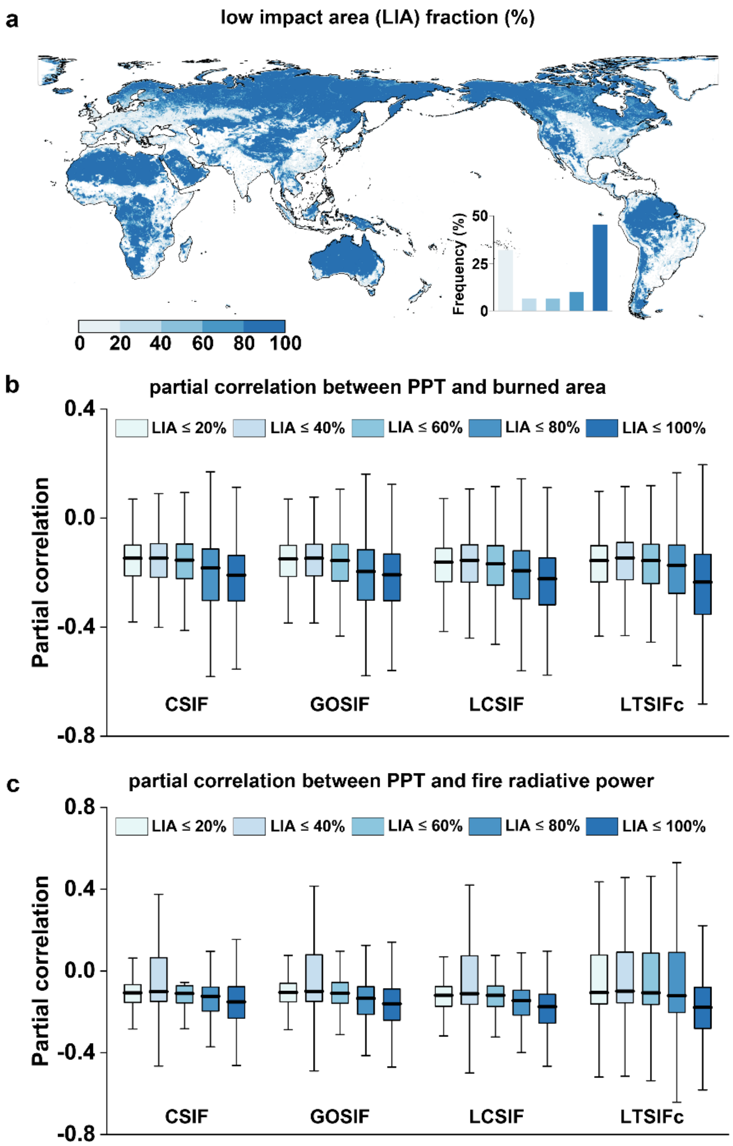

Supplementary Figure 24. The effects of peak photosynthesis timing (PPT) on fire activity (including burned area and fire radiative power) regulated by human activity. (a) Global map of low human impact area (LIA) fraction calculated as the percentage of 1-km LIA within the 0.25 °×0.25 °grid cell. Larger LIA indicates low-impact land by human, where fire activity may be mainly determined by natural factors. Lower LIA indicates areas with larger human impact, where fire activity may be largely influenced by human activity. (b) Partial correlation between PPT and burned area along the LIA gradients. (c) Same as (b) but for fire radiative power. We can see that in areas with lower human impact, PPT has larger effect on the subsequent burned area and fire radiative power. (审图号：GS 京 （2024）1632 号）

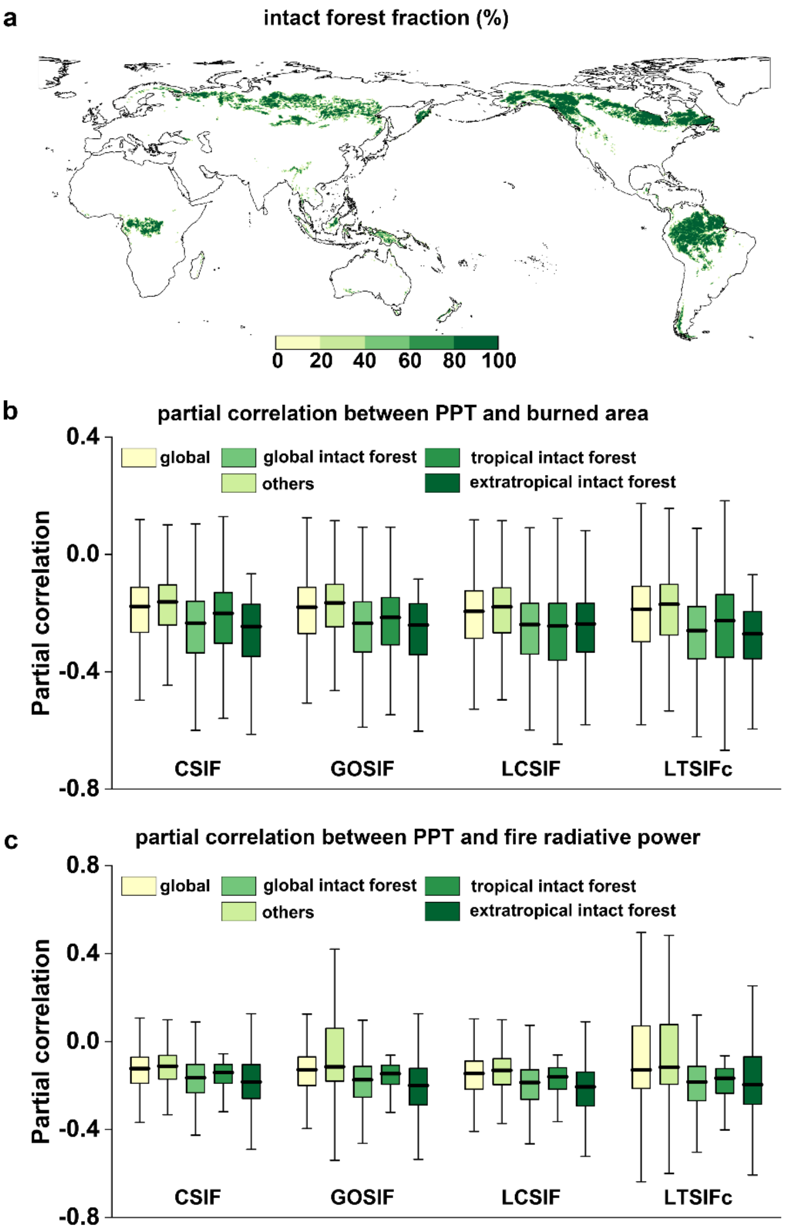

Supplementary Figure 25. The effects of peak photosynthesis timing (PPT) on fire activity (including burned area and fire radiative power) in intact forest. (a) Global map of intact forest fraction calculated as the percentage of intact forest within the 0.25°×0.25° grid cell. We consider intact forest as the area without human impact. (b) Comparisons of partial correlations between PPT and burned area among the globe, global intact forest, others (global areas outside of intact forest), tropical intact forest (25°S-25°N), and extratropical intact forest. (c) Same as (b) but for fire radiative power. We can see that in intact forest, PPT has larger effect on the subsequent burned area and fire radiative power. (审图号：GS 京 （2024）1632 号)

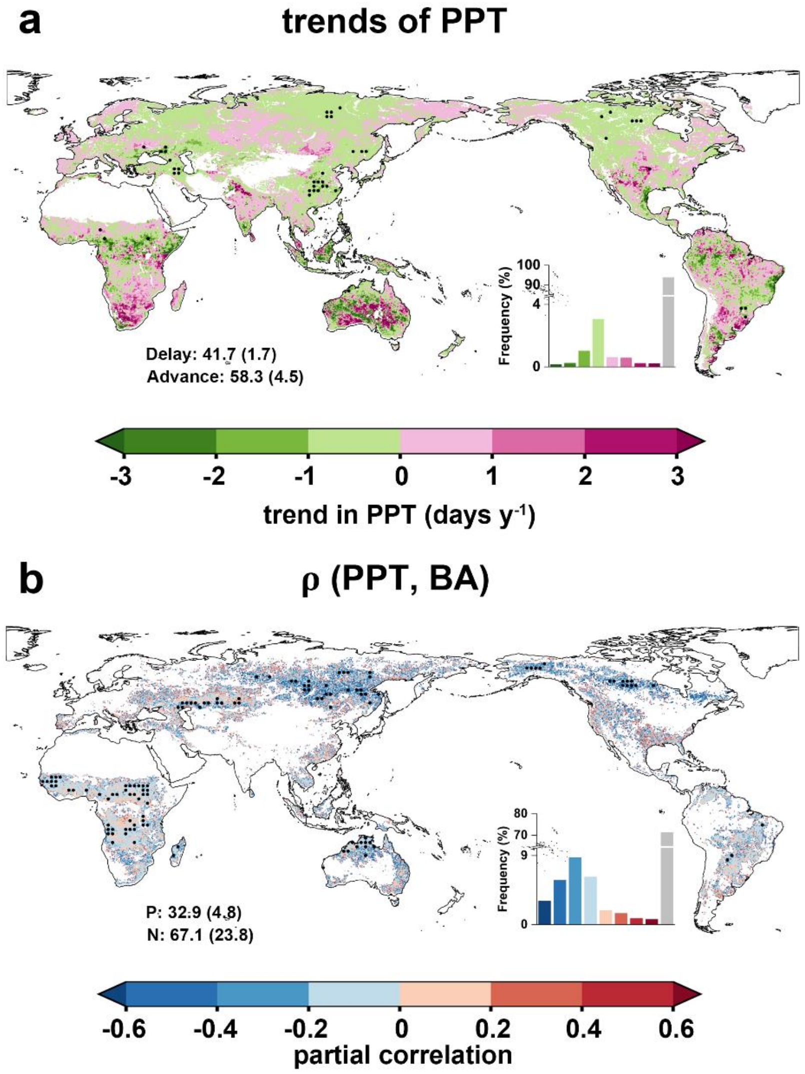

Supplementary Figure 26. Trend in peak photosynthesis timing (PPT) and partial correlation between PPT and the subsequent burned area at a 0.05 ° spatial scale. The black dots indicate regions with significant PPT trends and correlations (p-value < 0.05). The labels in (a) indicate the percentage of areas with delayed and advanced PPT, respectively. P and N indicate the percentage of positive and negative correlations, respectively. This analysis was based on CSIF-derived PPT and MODIS-derived global burned areas at a 0.05° spatial resolution. (审图号：GS 京 （2024） 1632 号）

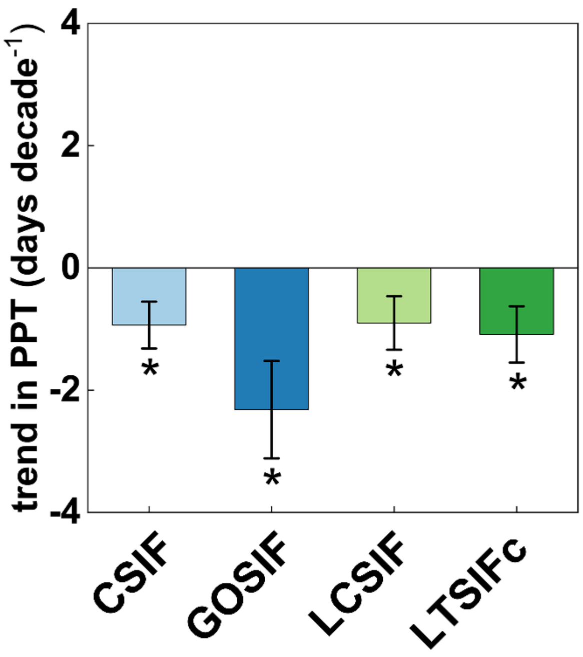

Supplementary Figure 27. Decadal trends in peak photosynthesis timing (PPT) over 2002-2018 derived from CSIF, GOSIF, LCSIF, and LT_SIFc for the northern ecosystems (>30 °N). Bar and error bar indicate decadal trend and corresponding 95% confidence interval, respectively, based on linear regression (see *Extraction of global peak photosynthesis timing* for detail). The asterisk denotes p-value < 0.05. We found that the trend of PPT in our study was consistent with previous studies that focused on the northern ecosystems, which demonstrates that our method used to calculate PPT is feasible and reasonable.

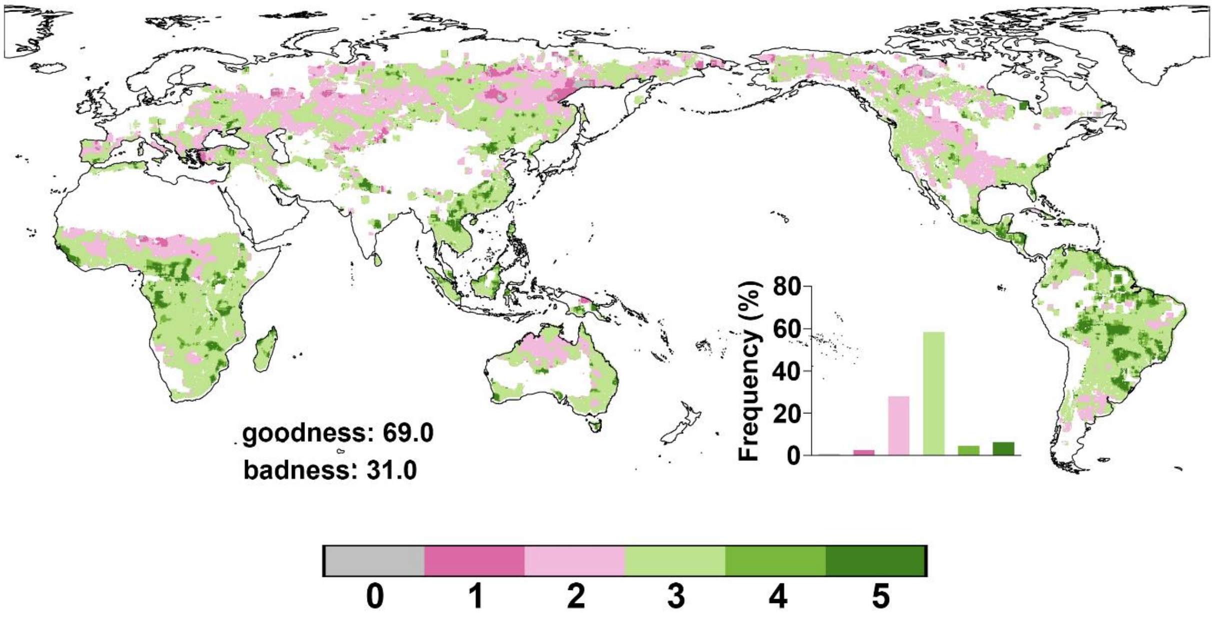

Supplementary Figure 28. Goodness of fit of the model using structural equation model (SEM) for CSIF-derived peak photosynthesis timing (PPT) and MODIS-derived burned area. We selected five metrics to evaluate the goodness of fitted model, i.e. GFI, CFI, RMSEA, NNFI, and SRMR. 0-5 indicate the number of criteria satisfied, and the model was considered reliable when three out of five criteria are met (green). (审图号：GS 京 （2024）1632 号）

Supplementary Tables

Supplementary Table 1. Descriptions and access information of the datasets.

Variables	Datasets	Resolution	Periods	Data access
SIF	CSIF	0.05°, 4-day	2001-2018	https://doi.org/10.17605/OSF.IO/8XQY6
	GOSIF	0.05°, 8-day	2001-2018	https://globalecology.unh.edu/data/GOSIF.html
	LCSIF	0.05°, bi-monthly	2001-2018	https://zenodo.org/records/7916879
	LT_SIFc	0.05°, monthly	2001-2018	https://doi.org/10.6084/m9.figshare.21546066.v1
NDVI	PKU GIMMS NDVI	1/12°, half-monthly	2001-2018	https://zenodo.org/records/8253971
Fire activity	NBAC	shapefile	2002-2018	https://cwfis.cfs.nrcan.gc.ca/datamart
	MTBS	shapefile	2002-2018	https://www.mtbs.gov/direct-download
	MCD64A1 V6, BA	500m, monthly	2001-2018	https://doi.org/10.5067/MODIS/MCD64A1.006
	MCD14ML V6, FRP	shapefile	2001-2018	https://www.earthdata.nasa.gov/learn/find-data/near-real-time/firms/mcd14ml
Climate variable	ERA5-Land monthly averaged reanalysis data (T, PRE, SM, Td, RAD)	0.1°, monthly	2001-2018	https://cds.climate.copernicus.eu/cdsapp#!/dataset/reanalysis-era5-land-monthly-means?tab=overview
CWD	TerraClimate	1/24°, monthly	2001-2018	https://www.climatologylab.org/terraclimate.html
BUI	Fire danger indices historical data	0.25°, daily	2001-2018	https://cds.climate.copernicus.eu/cdsapp#!/dataset/cems-fire-historical?tab=overview
Land cover type	MCD12Q1 V6 IGBP classification	500 m, yearly	2001-2018	https://doi.org/10.5067/MODIS/MCD12Q1.006
Climate classification	Köppen-Geiger climate classification	0.083°	present-day (1980-2016)	http://www.gloh2o.org/koppen/

Biome	Terrestrial Ecoregions of the World	shapefile	--	https://www.worldwildlife.org/publications/terrestrial-ecoregions-of-the-world
LIA	Low human impact area dataset	1 km	--	https://doi.org/10.5061/dryad.z612jm67g
Intact forest	Intact forest landscapes	shapefile	year 2000	https://intactforests.org/
NPP	The dataset of simulated daily net primary productivity over the globe during 1981-2019	0.072727°, daily	2001-2018	http://www.nesdc.org.cn/sdo/detail?id=612f42ee7e28172cbed3d80b

SIF: solar-induced chlorophyll fluorescence; NDVI: normalized difference vegetation index; BA: burned area; FRP: fire radiative power; T: 2 m air temperature; PRE: total precipitation; SM: soil moisture content; Td: 2 m dewpoint temperature; RAD: surface solar radiation downwards; CWD: climatic water deficit; BUI: build up index. T and Td were used to calculate vapor pressure deficit (VPD) and relative humidity (RH); LIA: low impact area; NPP: net primary productivity.

Supplementary Table 2. Summary of the FireMIP fire-vegetation models [41, 54] used in this study.

Model	Resolution	Period	Output
CLM [65]	2.5°×1.9°, monthly	2001-2012	GPP, BF
JSBACH-SPITFIRE [66]	1.875°×1.875°, monthly	2001-2012	GPP, BF
LPJ-GUESS-SPITFIRE [67]	0.5°×0.5°, monthly	2001-2012	GPP, BF
ORCHIDEE-SPITFIRE [68]	0.5°×0.5°, monthly	2001-2012	GPP BF
CTEM [69]	2.8125°×2.8125°, monthly	2001-2012	GPP, BF
JULES-INFERNO [70]	1.875°×1.245°, monthly	2001-2012	GPP, BF
LPJ-GUESS-SIMFIRE-BLAZE [71]	0.5°×0.5°, monthly	2001-2012	GPP, BF

GPP: gross primary productivity; BF: burned fraction, the fraction of burned area within a grid cell (%). Notably another two FireMIP models, LPJ-GUESS-GlobFIRM [72] and MC2 [73], were excluded in this study because they perform less well globally than other models in simulating global burned areas [41] and provide yearly burned area data.

References

1. Guanter L, Zhang Y, Jung M *et al.* Global and time-resolved monitoring of crop photosynthesis with chlorophyll fluorescence. *Proc Natl Acad Sci USA* 2014; **111**: E1327-33.
2. Frankenberg C, Fisher JB, Worden J *et al.* New global observations of the terrestrial carbon cycle from GOSAT: patterns of plant fluorescence with gross primary productivity. *Geophys Res Lett* 2011; **38**: L17706.
3. Joiner J, Yoshida Y, Vasilkov AP *et al.* First observations of global and seasonal terrestrial chlorophyll fluorescence from space. *Biogeosciences* 2011; **8**: 637-51.
4. Baker NR. Chlorophyll fluorescence: a probe of photosynthesis in vivo. *Annu Rev Plant Biol* 2008; **59**: 89-113.
5. Guanter L, Frankenberg C, Dudhia A *et al.* Retrieval and global assessment of terrestrial chlorophyll fluorescence from GOSAT space measurements. *Remote Sens Environ* 2012; **121**: 236-51.
6. Zhang Z, Zhang Y, Zhang Y. Generating high-resolution total canopy SIF emission from TROPOMI data: algorithm and application. *Remote Sens Environ* 2023; **295**: 113699.
7. Chen A, Meng F, Mao J *et al.* Photosynthesis phenology, as defined by solar-induced chlorophyll fluorescence, is overestimated by vegetation indices in the extratropical Northern Hemisphere. *Agric For Meteorol* 2022; **323**: 109027.
8. Huang Z, Zhou L, Chi Y. Spring phenology rather than climate dominates the trends in peak of growing season in the Northern Hemisphere. *Glob Chang Biol* 2023; **29**: 4543-55.
9. Yang J, Xiao X, Doughty R *et al.* TROPOMI SIF reveals large uncertainty in estimating the end of plant growing season from vegetation indices data in the Tibetan Plateau. *Remote Sens Environ* 2022; **280**: 113209.
10. Zhang Y, Joiner J, Alemohammad SH *et al.* A global spatially contiguous solar-induced fluorescence (CSIF) dataset using neural networks. *Biogeosciences* 2018; **15**: 5779-800.
11. Li X, Xiao J. A global, 0.05-degree product of solar-induced chlorophyll fluorescence derived from OCO-2, MODIS, and reanalysis data. *Remote Sens* 2019; **11**: 517.
12. Fang J, Lian X, Ryu Y *et al*. Reconstructed of a long-term spatially contiguous solar-induced fluorescence (LCSIF) over 1982-2022. arXiv: 2311.14987.
13. Wang S, Zhang Y, Ju W *et al.* Temporally corrected long-term satellite solar-induced fluorescence leads to improved estimation of global trends in vegetation photosynthesis during 1995–2018. *ISPRS J Photogramm Remote Sens* 2022; **194**: 222-34.
14. Wang X, Wu C. Estimating the peak of growing season (POS) of China's terrestrial ecosystems. *Agric For Meteorol* 2019; **278**: 107639.
15. Chen JM, Feng D, Mingzhen C. Locally adjusted cubic-spline capping for reconstructing seasonal trajectories of a satellite-derived surface parameter. *IEEE Trans Geosci Remote Sens* 2006; **44**: 2230-38.
16. Park T, Chen C, Macias-Fauria M *et al.* Changes in timing of seasonal peak photosynthetic activity in northern ecosystems. *Glob Chang Biol* 2019; **25**: 2382-95.
17. Gonsamo A, Chen JM, Ooi YW. Peak season plant activity shift towards spring is reflected

by increasing carbon uptake by extratropical ecosystems. *Glob Chang Biol* 2018; **24**: 2117-28.

18. Hall RJ, Skakun RS, Metsaranta JM *et al.* Generating annual estimates of forest fire disturbance in Canada: the National Burned Area Composite. *Int J Wildland Fire* 2020; **29**: 878-91.
19. Picotte JJ, Bhattarai K, Howard D *et al.* Changes to the Monitoring Trends in Burn Severity program mapping production procedures and data products. *Fire Ecol* 2020; **16**: 16.
20. Radeloff VC, Helmers DP, Kramer HA *et al.* Rapid growth of the US wildland-urban interface raises wildfire risk. *Proc Natl Acad Sci USA* 2018; **115**: 3314-19.
21. Picotte JJ, Peterson B, Meier G *et al.* 1984–2010 trends in fire burn severity and area for the conterminous US. *Int J Wildland Fire* 2016; **25**: 413-20.
22. Metsaranta JM, Hudson B, Smyth C *et al.* Future fire risk and the greenhouse gas mitigation potential of forest rehabilitation in British Columbia, Canada. *For Ecol Manage* 2023; **529**: 120729.
23. Mansuy N, Miller C, Parisien M-A *et al.* Contrasting human influences and macro-environmental factors on fire activity inside and outside protected areas of North America. *Environ Res Lett* 2019; **14**: 064007.
24. Giglio L, Boschetti L, Roy DP *et al.* The Collection 6 MODIS burned area mapping algorithm and product. *Remote Sens Environ* 2018; **217**: 72-85.
25. Jain P, Castellanos-Acuna D, Coogan SCP *et al.* Observed increases in extreme fire weather driven by atmospheric humidity and temperature. *Nat Clim Chang* 2022; **12**: 63-70.
26. Descals A, Gaveau DLA, Verger A *et al.* Unprecedented fire activity above the Arctic Circle linked to rising temperatures. *Science* 2022; **378**: 532-7.
27. Vitolo C, Di Giuseppe F, Barnard C *et al.* ERA5-based global meteorological wildfire danger maps. *Sci Data* 2020; **7**: 216.
28. Yuan W, Zheng Y, Piao S *et al.* Increased atmospheric vapor pressure deficit reduces global vegetation growth. *Sci Adv* 2019; **5**: eaax1396.
29. Muñoz-Sabater J, Dutra E, Agustí-Panareda A *et al.* ERA5-Land: a state-of-the-art global reanalysis dataset for land applications. *Earth Syst Sci Data* 2021; **13**: 4349-83.
30. Abatzoglou JT, Dobrowski SZ, Parks SA *et al.* TerraClimate, a high-resolution global dataset of monthly climate and climatic water balance from 1958-2015. *Sci Data* 2018; **5**: 170191.
31. Luo K, Wang X, de Jong M *et al.* Drought triggers and sustains overnight fires in North America. *Nature* 2024; **627**: 321-7.
32. Jolly WM, Cochrane MA, Freeborn PH *et al.* Climate-induced variations in global wildfire danger from 1979 to 2013. *Nat Commun* 2015; **6**: 7537.
33. Abatzoglou JT, Williams AP, Barbero R. Global emergence of anthropogenic climate change in fire weather indices. *Geophys Res Lett* 2019; **46**: 326-36.
34. Touma D, Stevenson S, Lehner F *et al.* Human-driven greenhouse gas and aerosol emissions cause distinct regional impacts on extreme fire weather. *Nat Commun* 2021; **12**: 212.
35. Freeborn PH, Wooster MJ, Roy DP *et al.* Quantification of MODIS fire radiative power (FRP)

measurement uncertainty for use in satellite-based active fire characterization and biomass burning estimation. *Geophys Res Lett* 2014; **41**: 1988-94.

36. Giglio L, Schroeder W, Justice CO. The collection 6 MODIS active fire detection algorithm and fire products. *Remote Sens Environ* 2016; **178**: 31-41.
37. Chen JM, Ju W, Ciais P *et al.* Vegetation structural change since 1981 significantly enhanced the terrestrial carbon sink. *Nat Commun* 2019; **10**: 4259.
38. Forkel M, Andela N, Harrison SP *et al.* Emergent relationships with respect to burned area in global satellite observations and fire-enabled vegetation models. *Biogeosciences* 2019; **16**: 57-76.
39. Bistinas I, Harrison SP, Prentice IC *et al.* Causal relationships versus emergent patterns in the global controls of fire frequency. *Biogeosciences* 2014; **11**: 5087-5101.
40. Rabin SS, Melton JR, Lasslop G *et al.* The Fire Modeling Intercomparison Project (FireMIP), phase 1: experimental and analytical protocols with detailed model descriptions. *Geosci Model Dev* 2017; **10**: 1175-97.
41. Hantson S, Kelley DI, Arneth A *et al.* Quantitative assessment of fire and vegetation properties in simulations with fire-enabled vegetation models from the Fire Model Intercomparison Project. *Geosci Model Dev* 2020; **13**: 3299-318.
42. Beck HE, Zimmermann NE, McVicar TR *et al.* Present and future Koppen-Geiger climate classification maps at 1-km resolution. *Sci Data* 2018; **5**: 180214.
43. Olson DM, Dinerstein E, Wikramanayake ED *et al.* Terrestrial ecoregions of the world: a new map of life on earth: a new global map of terrestrial ecoregions provides an innovative tool for conserving biodiversity. *BioScience* 2001; **51**: 933-8.
44. Li M, Cao S, Zhu Z *et al.* Spatiotemporally consistent global dataset of the GIMMS Normalized Difference Vegetation Index (PKU GIMMS NDVI) from 1982 to 2022. *Earth Syst Sci Data* 2023; **15**: 4181-203.
45. Forzieri G, Miralles DG, Ciais P *et al.* Increased control of vegetation on global terrestrial energy fluxes. *Nat Clim Chang* 2020; **10**: 356-62.
46. Seneviratne SI, Corti T, Davin EL *et al.* Investigating soil moisture–climate interactions in a changing climate: a review. *Earth Sci Rev* 2010; **99**: 125-61.
47. Gao S, Liang E, Liu R *et al.* An earlier start of the thermal growing season enhances tree growth in cold humid areas but not in dry areas. *Nat Ecol Evol* 2022; **6**: 397-404.
48. Xie F, Yuan N, Qi Y *et al.* Is long-term climate memory important in temperature/precipitation predictions over China? *Theor Appl Climatol* 2019; **137**: 459-66.
49. Yuan N, Huang Y, Duan J *et al.* On climate prediction: how much can we expect from climate memory? *Clim Dyn* 2019; **52**: 855-64.
50. Danabasoglu G, Lamarque JF, Bacmeister J *et al.* The Community Earth System Model version 2 (CESM2). *J Adv Model Earth Syst* 2020; **12**(2): e2019MS001916.
51. Lawrence PJ, Chase TN. Representing a new MODIS consistent land surface in the Community Land Model (CLM 3.0). *J Geophys Res* 2007; **112**: G01023.
52. Myneni RB, Hoffman S, Knyazikhin Y *et al.* Global products of vegetation leaf area and

fraction absorbed PAR from year one of MODIS data. *Remote Sens Environ* 2002; **83**: 214-31.

53. Zeng X, Shaikh M, Dai Y *et al.* Coupling of the Common Land Model to the NCAR Community Climate Model. *J Clim* 2002; **15**: 1832-54.
54. Hantson S, Rabin S, Kelley DI *et al.* Model outputs: quantitative assessment of fire and vegetation properties in historical simulations with fire-enabled vegetation models from the Fire Model Intercomparison Project [Data set]. Zenodo; 2019.
55. Besnard S, Santoro M, Cartus O *et al.* Global sensitivities of forest carbon changes to environmental conditions. *Glob Chang Biol* 2021; **27**: 6467-83.
56. Li W, Migliavacca M, Forkel M *et al.* Revisiting global vegetation controls using multi-layer soil moisture. *Geophys Res Lett* 2021; **48**: e2021GL092856.
57. Li W, Migliavacca M, Forkel M *et al.* Widespread increasing vegetation sensitivity to soil moisture. *Nat Commun* 2022; **13**: 3959.
58. Bowman DMJS, Kolden CA, Abatzoglou JT *et al.* Vegetation fires in the Anthropocene. *Nat Rev Earth Environ* 2020; **1**: 500-15.
59. Andela N, Morton DC, Giglio L *et al.* A human-driven decline in global burned area. *Science* 2017; **356**: 1356-62.
60. Balch JK, Bradley BA, Abatzoglou JT *et al.* Human-started wildfires expand the fire niche across the United States. *Proc Natl Acad Sci USA* 2017; **114**: 2946-51.
61. Janssen TAJ, Jones MW, Finney D *et al.* Extratropical forests increasingly at risk due to lightning fires. *Nat Geosci* 2023; **16**: 1136-44.
62. Lapola DM, Pinho P, Barlow J *et al.* The drivers and impacts of Amazon forest degradation. *Science* 2023; **379**: eabp8622.
63. Jacobson AP, Riggio J, A MT *et al.* Global areas of low human impact ('Low Impact Areas') and fragmentation of the natural world. *Sci Rep* 2019; **9**: 14179.
64. Potapov P, Hansen MC, Laestadius L *et al.* The last frontiers of wilderness: tracking loss of intact forest landscapes from 2000 to 2013. *Sci Adv* 2017; **3**: e1600821.
65. Li F, Levis S, Ward DS. Quantifying the role of fire in the Earth system – Part 1: improved global fire modeling in the Community Earth System Model (CESM1). *Biogeosciences* 2013; **10**: 2293-314.
66. Lasslop G, Thonicke K, Kloster S. SPITFIRE within the MPI Earth system model: model development and evaluation. *J Adv Model Earth Syst* 2014; **6**: 740-55.
67. Lehsten V, Tansey K, Balzter H *et al.* Estimating carbon emissions from African wildfires. *Biogeosciences* 2009; **6**: 349-60.
68. Yue C, Ciais P, Cadule P *et al.* Modelling the role of fires in the terrestrial carbon balance by incorporating SPITFIRE into the global vegetation model ORCHIDEE – Part 1: simulating historical global burned area and fire regimes. *Geosci Model Dev* 2014; **7**: 2747-67.
69. Melton JR, Arora VK. Competition between plant functional types in the Canadian Terrestrial Ecosystem Model (CTEM) v. 2.0. *Geosci Model Dev* 2016; **9**: 323-61.
70. Mangeon S, Voulgarakis A, Gilham R *et al.* INFERNO: a fire and emissions scheme for the

UK Met Office's Unified Model. *Geosci Model Dev* 2016; **9**: 2685-700.

71. Knorr W, Jiang L, Arneth A. Climate, CO_2 and human population impacts on global wildfire emissions. *Biogeosciences* 2016; **13**: 267-82.
72. Smith B, Wårlind D, Arneth A *et al.* Implications of incorporating N cycling and N limitations on primary production in an individual-based dynamic vegetation model. *Biogeosciences* 2014; **11**: 2027-54.
73. Bachelet D, Ferschweiler K, Sheehan TJ *et al.* Projected carbon stocks in the conterminous USA with land use and variable fire regimes. *Glob Chang Biol* 2015; **21**: 4548-60.
